# Effects of music therapy on delirium, clinical outcomes, and psychological and sleep outcomes in adult ICU patients: a systematic review and meta-analysis

**DOI:** 10.3389/fmed.2026.1857001

**Published:** 2026-07-01

**Authors:** Ying Yuan, Sijing Wang, Hao Wang, Wensi Wang, Kaili Dai

**Affiliations:** 1 Department of Critical Care Medicine, West China School of Public Health and West China Fourth Hospital, Sichuan University, Chengdu, China; 2 Department of Dermatology, West China School of Public Health and West China Fourth Hospital, Sichuan University, Chengdu, China

**Keywords:** music therapy, intensive care units, delirium, clinical outcomes, meta-analysis

## Abstract

**Background:**

Delirium and psychological stress are major clinical challenges for adult ICU patients, contributing to increased mortality and resource consumption. This study evaluated the efficacy of music therapy (MT) on delirium incidence, resource utilization, and psychological and sleep outcomes.

**Methods:**

Seven electronic databases (Medline, Embase, Cochrane Library, CNKI, Wanfang, VIP, and CBM) were searched from inception to December 20, 2025. Following PRISMA guidelines, 15 randomized controlled trials (RCTs) involving 1,552 patients were included. Two researchers independently performed screening and data extraction. Risk of bias was assessed using RoB 2.0, and evidence certainty was graded using the GRADE system. Primary outcomes focused on delirium assessments; secondary outcomes included mortality and resource utilization; exploratory outcomes encompassed anxiety, depression, pain, and sleep.

**Results:**

A total of 863 records were identified, and 15 RCTs involving 1,552 patients were finally included. Meta-analysis indicated that music therapy significantly reduced the incidence of delirium (*RR* = 0.49; 95% *CI*: 0.40–0.60; *p* < 0.001). Regarding resource utilization, after excluding heterogeneous outliers through sensitivity analysis, the intervention group showed a significantly shorter duration of mechanical ventilation (*SMD* = −0.25; *p* = 0.009) and ICU length of stay (*MD* = −1.07 days; *p* = 0.004). However, there was no significant difference between the two groups in terms of hospital length of stay (*MD* = −1.37 days; 95% *CI*, −4.06 to 1.33; *p* = 0.32). For exploratory outcomes, depression symptoms were significantly improved (*SMD* = −1.30; *p* < 0.001); objective acute sleep quality (based on the RCSQ scale) was markedly enhanced (*SMD* = −0.62; *p* < 0.001, *I*^2^ = 0%); and anxiety was significantly relieved (*SMD* = −0.79; *p* = 0.02), with subgroup analysis suggesting a more pronounced effect in Chinese cohorts. No significant differences were observed in short-term mortality or pain scores. GRADE grading indicated high-quality evidence for delirium incidence and ICU length of stay.

**Conclusion:**

Music therapy is a safe, low-cost non-pharmacological intervention that significantly reduces delirium risk, restores sleep architecture, alleviates anxiety and depression, and accelerates weaning from mechanical ventilation in adult ICU patients. However, due to heterogeneity and lower certainty in some secondary outcomes, larger multicenter trials are needed before routinely integrating music therapy into standard ICU and ERAS protocols.

**Systematic review registration:**

https://www.crd.york.ac.uk/PROSPERO/view/CRD420261329297.

## 1 Introduction

Adult patients in the intensive care unit (ICU) frequently experience profound physiological and psychological stress, driven by critical illness, invasive life-support measures, and the highly complex treatment environment (1). As the most prevalent acute brain dysfunction syndrome in the ICU, delirium carries substantial clinical significance in this context. It is associated not only with increased short-term mortality but also with long-term cognitive impairment and a significant escalation in healthcare resource consumption (2, 3). While the clinical management of delirium and psychological stress in the ICU currently relies heavily on sedative and analgesic medications, these pharmacological approaches are often fraught with risks, such as respiratory depression and hemodynamic instability. Such complications can prolong the duration of mechanical ventilation and, paradoxically, induce iatrogenic delirium (4). Consequently, exploring safe and effective non-pharmacological adjunctive strategies has become a pivotal direction for optimizing the management of critically ill patients (5). As a promising non-pharmacological intervention, music therapy has been evaluated in previous meta-analyses; however, these have primarily focused on subjective improvements in patient-reported pain or anxiety (6–8). Although these findings are instructive, the underlying original studies often exhibit high heterogeneity and rarely use objective clinical endpoints, such as delirium incidence, as core evaluation metrics. This lack of objective focus makes it challenging to establish consistent, high-level evidence that can guide clinical decision-making.

To address these research gaps, the present study aims to systematically evaluate the impact of music therapy on objective clinical outcomes in adult ICU patients, with a primary focus on delirium incidence and short-term mortality. Furthermore, we will analyze secondary effects on healthcare resource utilization (e.g., duration of mechanical ventilation and ICU length of stay) and on psychological and sleep outcomes (e.g., anxiety and sleep quality). By employing rigorous sensitivity analyses and subgroup analyses to dissect sources of heterogeneity, this study seeks to provide higher-quality, more targeted evidence-based support for the formulation and optimization of non-pharmacological intervention strategies in the ICU.

## 2 Methods

### 2.1 Protocol and registration

The protocol for this systematic review and meta-analysis was prospectively registered with PROSPERO (Identifier: CRD420261329297). The study was conducted and reported in strict accordance with the PRISMA 2020 (Preferred Reporting Items for Systematic Reviews and Meta-Analyses) statement (9).

### 2.2 Data sources and search strategy

A comprehensive search was performed across seven electronic databases: Medline, Embase, and the Cochrane Library, as well as Chinese databases including CNKI, Wanfang, VIP, and CBM. The search strategies (Supplementary material 1) were designed and executed independently by research team members with extensive training in evidence-based medicine and systematic review methodologies. The search period spanned from database inception to December 20, 2025. A strategy combining Subject Headings (e.g., MeSH for Medline and Emtree for Embase) and free-text terms was employed. For instance, the Medline search string was: (“Delirium”[Mesh] OR “delirium”[Title/Abstract]) AND (“Intensive Care Units”[Mesh] OR “intensive care”[Title/Abstract]) AND (“Music Therapy”[Mesh] OR “music”[Title/Abstract]). Furthermore, reference lists of all eligible studies were manually screened to ensure a comprehensive literature retrieval. Inclusion and Exclusion Criteria Studies were selected based on the PICOS framework: Population (P): Adult ICU patients (aged ≥18 years); Intervention (I): Pure music therapy administered in addition to routine care; Comparator (C): Routine ICU care or environmental noise control alone; Outcomes (O): The primary outcomes focused on delirium-related clinical assessments, specifically the incidence of delirium, delirium-free days, and delirium severity. Secondary outcomes comprised survival prognosis (short-term mortality, total hospital length of stay) and resource utilization (duration of mechanical ventilation, ICU length of stay). Exploratory outcomes covered psychological and sleep indicators (anxiety, depression, pain, and sleep quality); Study Design (S): Randomized controlled trials (RCTs). Exclusion criteria included: studies involving mixed interventions (e.g., combining music with other non-pharmacological therapies), non-adult ICU populations, and studies with missing or unextractable data.

### 2.3 Data extraction and quality assessment

To ensure objectivity and rigor, two researchers trained in evidence-based medicine independently performed literature screening and data extraction. The two researchers then compared their individual screening results and extracted the data side by side to identify any inconsistencies. Extracted data included first author, publication year, country, sample size, baseline patient characteristics, and predefined outcome measures. The methodological quality of the included RCTs was assessed using the revised Cochrane risk-of-bias tool for randomized trials (RoB 2.0) (10). The overall risk of bias for each study was determined by the highest risk level identified in any single domain. Any disagreements were resolved through discussion; if consensus could not be reached, a third senior researcher was consulted for final adjudication.

### 2.4 Statistical analysis

Statistical analyses were performed using RevMan 5.4. Heterogeneity among studies was evaluated using the *I*^2^ statistic, where *I*^2^ ≥ 50% indicated substantial heterogeneity, prompting the use of a random-effects model and subsequent sensitivity analyses (11). To assess the potential for publication bias, visual inspection of funnel plots was complemented by Egger’s regression test (12). The certainty of evidence for each outcome was evaluated using the Grading of Recommendations Assessment, Development, and Evaluation (GRADE) framework. Evidence certainty was downgraded based on five domains: risk of bias, inconsistency, indirectness, imprecision, and publication bias. The overall certainty of the evidence for each specific outcome was classified into one of four levels: high, moderate, low, or very low (13).

## 3 Results

### 3.1 Literature search and study characteristics

**Figure 1:**
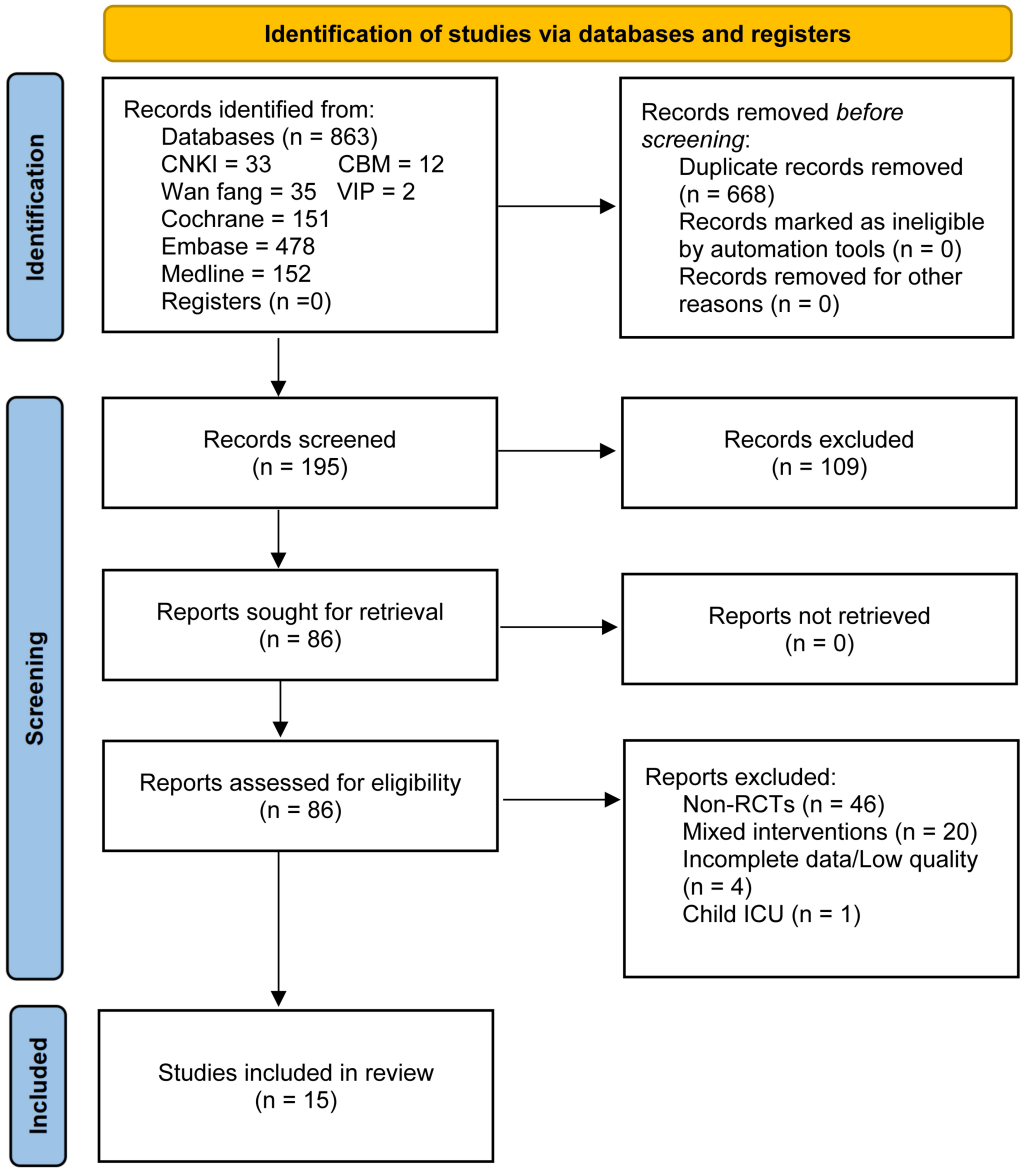
PRISMA 2020 flow diagram for the study selection process.

Of the 863 articles identified through systematic database and citation searching, 15 randomized clinical trials (RCTs) involving 1,552 participants met the final inclusion criteria (Figure 1). Baseline characteristics across the included studies were highly consistent and clinically representative (Table 1). The mean age of participants ranged from 37 to 75 years, and primary diagnoses included severe pneumonia, acute respiratory distress syndrome (ARDS), acute exacerbation of chronic obstructive pulmonary disease (COPD), and major cardiovascular surgery. Most participants required invasive mechanical ventilation, with APACHE II scores ranging from 10 to 22 at ICU admission, indicating severe disease.

**Table 1:** Baseline characteristics of the included studies.

Study (Year)	Country	Study design	Sample size (MT/UC)	Mean age (Years)	Sex ratio (Male/Female)	Interventions (Music type/Frequency)	Outcomes assessed
Zhang, 2017 (18)	China	RCT	116/115	57.68 ± 14.41	124/107	Patient’s choice (or Classical/Light/Pop/Religious)/20–40 min, 3 times/day	1
You, 2019 (32)	China	RCT	31/31	57.01 ± 10.55	45/17	Light music/20–40 min, 3 times/day	1, 4, 6, 7, 8
Khan, 2020 (14)	USA	RCT	34/18	57.40 ± 14.20	25/27	Personalized or slow-tempo music/60 min, 2 times/day (up to 7 days)	2, 7, 10
Esfahanian, 2022 (15)	Iran	RCT	100/100	63.45 ± 7.78	160/40	Relaxation music (new-age genre, nature sounds)/60 min, 2 times/day (for 7 days)	1
Kuang, 2022 (33)	China	RCT	30/30	54.77 ± 4.11	33/27	Soothing music/30 min before sleep (1 time/day)	1, 7, 8, 9
Yue, 2023 (23)	China	RCT	57/64	72.92 ± 11.19	65/56	Five-element music (based on meridian clock)/30 min, 2 times/day	1, 6, 9
Yin, 2023 (31)	China	RCT	52/52	52.75 ± 13.75	101/3	Patient’s choice (relaxing)/30 min before and after endotracheal suction	1, 3, 5, 6, 10
Kakar, 2023 (16)	Netherlands	RCT	42/42	62.74 ± 10.28	62/32	Patient’s preferred music/≥ 30 min, 2 times/day for 3 days	1, 3, 4, 5, 6, 7, 9, 10
Dallı, 2023 (22)	Turkey	RCT	12/12	54.50 ± 13.50	12/12	MusiCure (relaxing, 60–80 bpm)/60 min, 2 times/day for 5 days	2, 7, 10
Tao, 2024 (21)	China	RCT	50/50	40.36 ± 3.46	64/36	Patient’s choice (light music, folk, opera, pop)/30 min per session	1, 5, 6, 9
Wei, 2024 (19)	China	RCT	40/32	37.16 ± 4.75	42/30	Five-Element/30–60 min, 3 times/day	1, 3, 6
Lin, 2024 (24)	China	RCT	63/69	67.92 ± 17.02	73/59	Five-Element (Ziwu Liuzhu)/30 min, 2 times/day	1, 6, 7, 8, 9
Liu, 2025 (25)	China	RCT	65/64	61.49 ± 10.84	81/48	Light music/30 min, 3 times/day	1, 5, 6, 10
Khan, 2025 (17)	USA	RCT	79/79	68.00 ± 9.20	86/72	Slow-tempo music (60–80 bpm)/60 min, 2 times/day (up to 7 days)	2, 3, 4, 5, 6, 7, 10
Culshaw, 2025 (20)	USA	RCT	6/7	65.8 ± 9.00	6/7	Commercial or preferred music during SAT/up to 90 min, 1 time/day (for up to 3 days)	2

(1) MT, Music Therapy; UC, Usual Care; RCT, Randomized Controlled Trial; NR, Not Reported. (2) Outcomes: 1 = Incidence of delirium; 2 = Coma-free days/Delirium severity; 3 = Short-term mortality; 4 = Hospital length of stay; 5 = Duration of mechanical ventilation; 6 = ICU length of stay; 7 = Anxiety; 8 = Depression; 9 = Sleep quality; 10 = CPOT score. (3) For the study by Yin 2023, the baseline sex ratio was extracted using the raw absolute frequencies (*n*) provided by the authors, as the corresponding percentages reported in their original publication contained apparent typographical/calculation errors. (4) For Dallı 2023, which utilized a 3-arm design (music vs. noise reduction vs. standard care), baseline data were extracted specifically for the music therapy and standard care groups for pairwise comparison.

### 3.2 Risk of bias assessment

**Figure 2:**
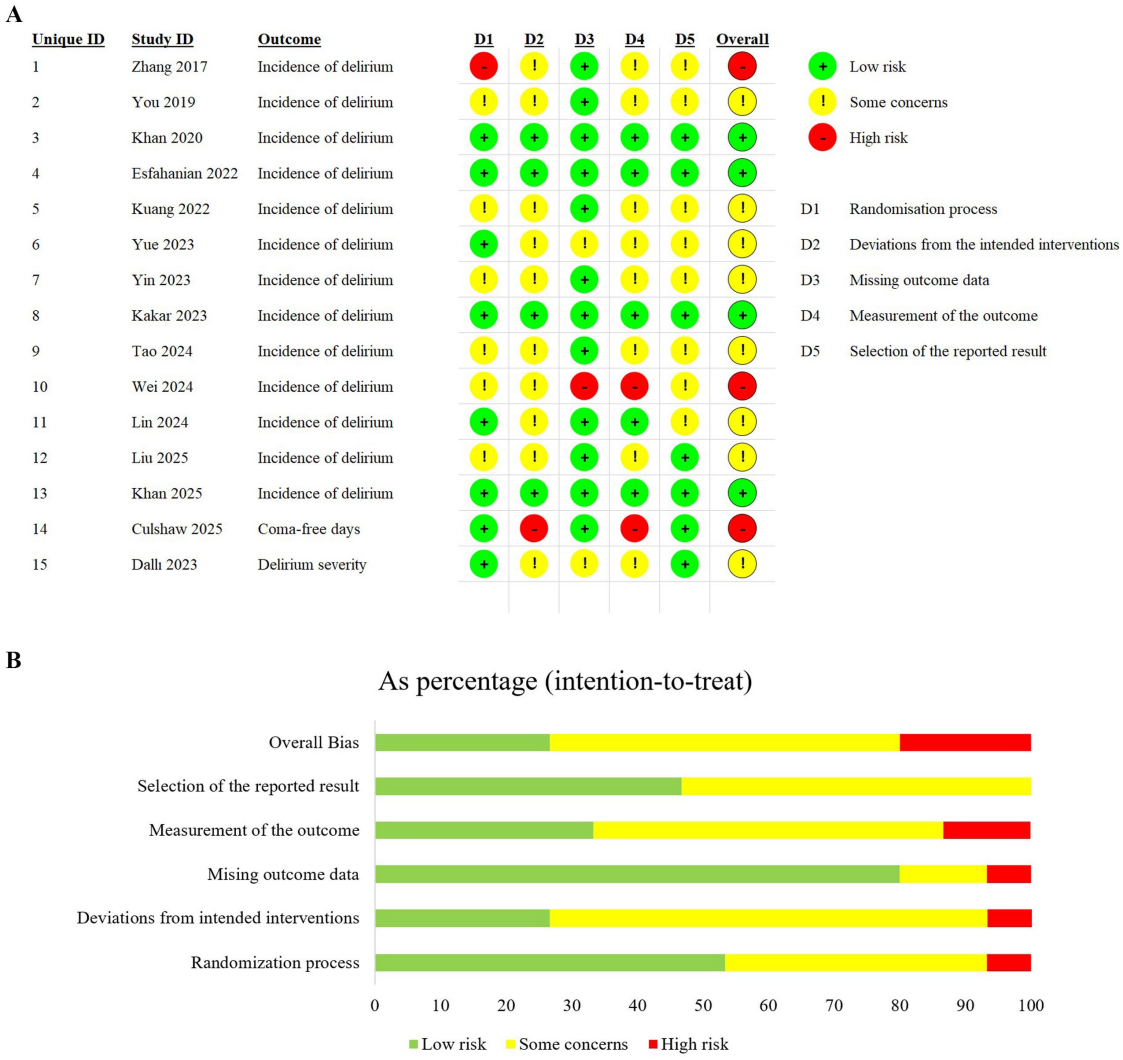
Risk of bias assessment for delirium-related outcomes. **(A)** Traffic light plot of risk of bias for each included study. **(B)** Risk of bias summary presented as percentages across all domains.

The methodological quality of the 15 included RCTs was independently evaluated using the Revised Cochrane Risk-of-Bias tool for randomized trials (RoB 2.0). The summary of the assessment for delirium-related outcomes is presented in Figure 2. As illustrated in the summary plot, 4 studies (26.7%) (14–17) were rated as having a low overall risk of bias. Eight studies (53.3%) were categorized as having some concerns, while 3 studies (20.0%) (18–20) were assessed as high risk.

#### 3.2.1 Randomization process (D1)

Approximately 53.3% (*n* = 8) of the trials demonstrated a low risk of bias, employing appropriate random sequence generation and allocation concealment. Only one study (6.7%) was rated as high risk due to unclear randomization procedures.

#### 3.2.2 Deviations from intended interventions (D2)

This domain posed the most significant challenge, with 66.7% (*n* = 10) of studies rated as having some concerns. This was primarily due to the inherent difficulty of blinding participants and personnel in nursing interventions like music therapy. Only 4 studies (26.7%) achieved a low risk rating here.

#### 3.2.3 Missing outcome data (D3)

The majority of the studies (80.0%, n = 12) demonstrated a low risk of bias in this domain. Two studies (13.3%) were assessed as having some concerns due to high attrition rates where the potential impact of missing data on the estimated effect could not be entirely ruled out. Only one study (6.7%) (19) was rated as high risk due to substantial missing data.

#### 3.2.4 Measurement of the outcome (D4)

High risk was identified in 2 studies (13.3%) (19, 20). For others, the risk was often rated as “some concerns” because outcome assessors were aware of the group assignments, even when standardized tools like CAM-ICU-7 were used.

#### 3.2.5 Selection of the reported result (D5)

No studies were at high risk in this domain. Seven studies (46.7%) were rated as low risk, typically supported by the availability of pre-registered clinical trial protocols or published statistical analysis plans. The remaining 8 studies (53.3%) were assessed as having some concerns, mainly due to the lack of accessible protocols to confirm the predefined analysis strategy.

### 3.3 Primary outcomes: delirium

**Figure 3:**
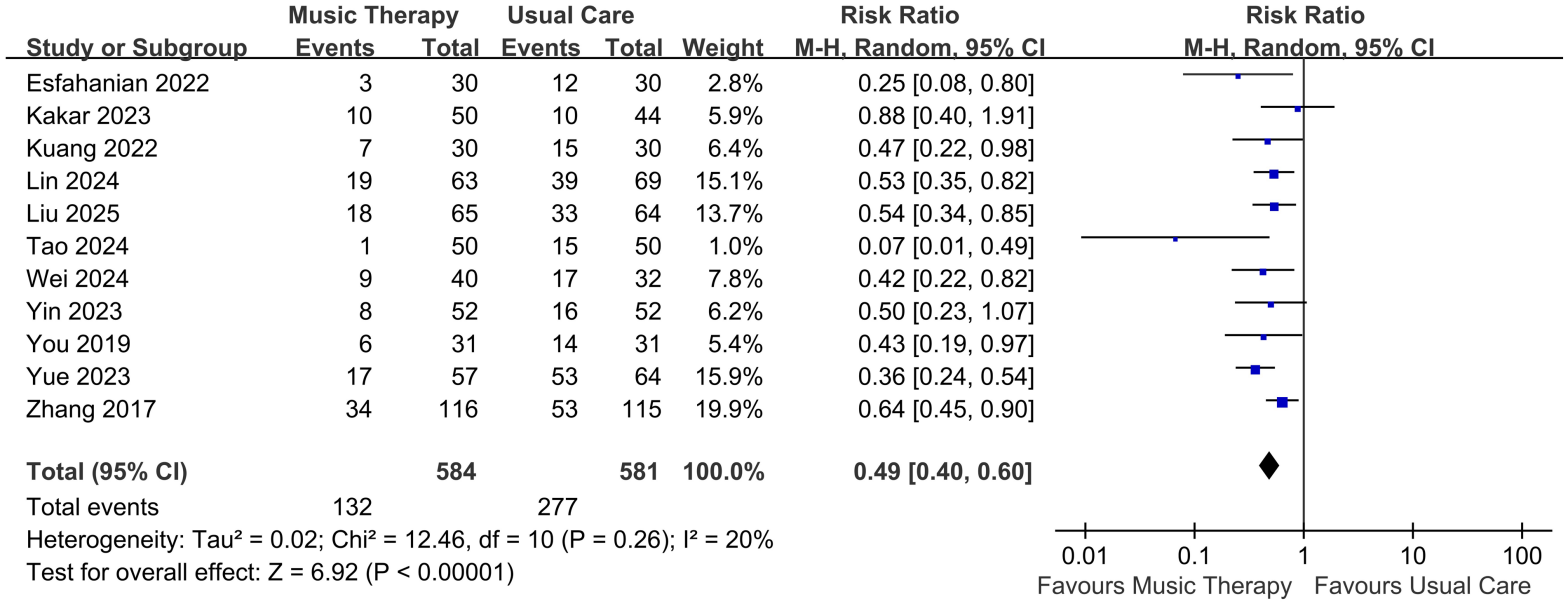
Forest plot comparing the incidence of delirium between the music therapy and usual care groups.

A meta-analysis observed a significant reduction in the incidence of delirium with music therapy (*RR* = 0.49; 95% *CI*, 0.40 to 0.60; *p* < 0.00001; *I*^2^ = 20%) (Figure 3). Sensitivity analyses were conducted by identifying and excluding potential outliers; after removing a clinical outlier with an extreme effect size (21), heterogeneity was eliminated (*I*^2^ = 0%), and the pooled effect remained significant. Regarding delirium-free days, after excluding a small-sample study (20), heterogeneity decreased to 55%, with no significant difference observed between groups (*SMD* = 0.14).

### 3.4 Secondary outcomes: clinical and resource utilization

**Figure 4:**
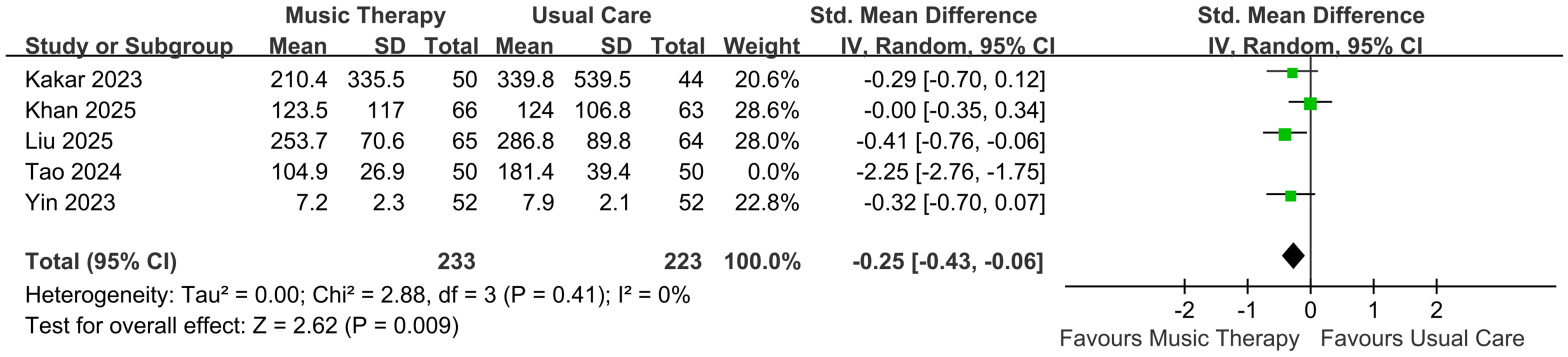
Forest plot for the duration of mechanical ventilation after sensitivity analysis.

**Figure 5:**
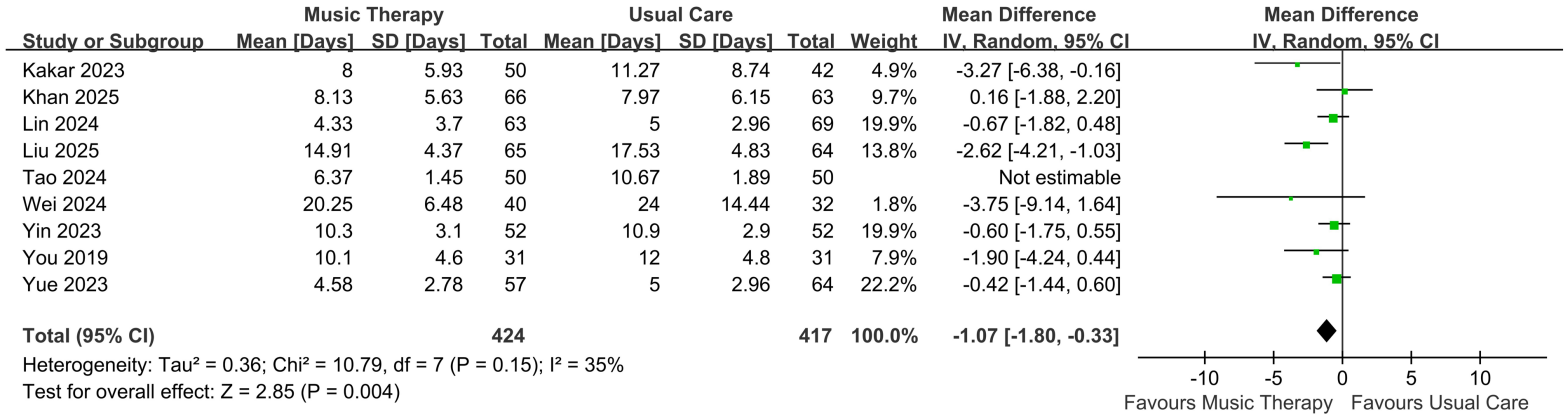
Forest plot for intensive care unit (ICU) length of stay after sensitivity analysis.

**Figure 6:**
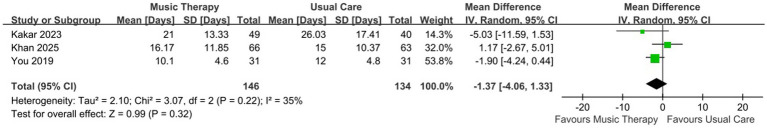
Forest plot for hospital length of stay (LOS).

Regarding medical resource utilization, the initial analysis of mechanical ventilation duration showed high heterogeneity (*I*^2^ = 93%). However, sensitivity analysis excluding clinical outliers completely resolved this heterogeneity (*I*^2^ = 0%) (Figure 4). The results demonstrated that the music therapy group had a significantly shorter duration of mechanical ventilation (*SMD* = −0.25; 95% *CI*, −0.43 to −0.06; *p* = 0.009) and a significantly reduced ICU length of stay (*MD* = −1.07; *p* = 0.004) compared with the usual care group (Figure 5). Additionally, the meta-analysis of three studies reported on hospital length of stay (LOS). The pooled results indicated that music therapy did not significantly reduce the hospital LOS compared to usual care (*MD* = −1.37 days; 95% *CI*, −4.06 to 1.33; *p* = 0.32), with low heterogeneity (*I*^2^ = 35%; *p* = 0.22) (Figure 6).

### 3.5 Exploratory outcomes: psychology, sleep, and pain

**Figure 7:**
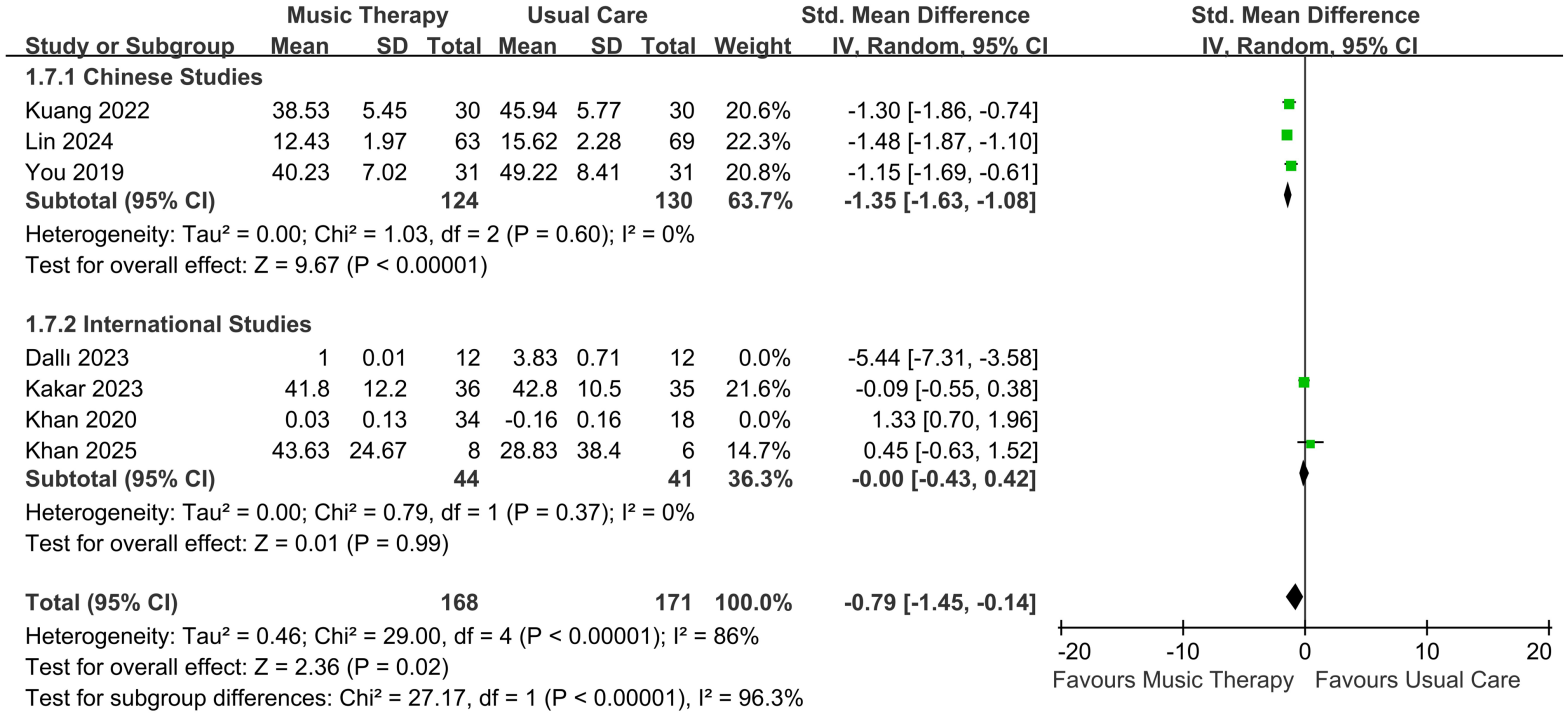
Forest plot of sensitivity and subgroup analysis for anxiety.

**Figure 8:**
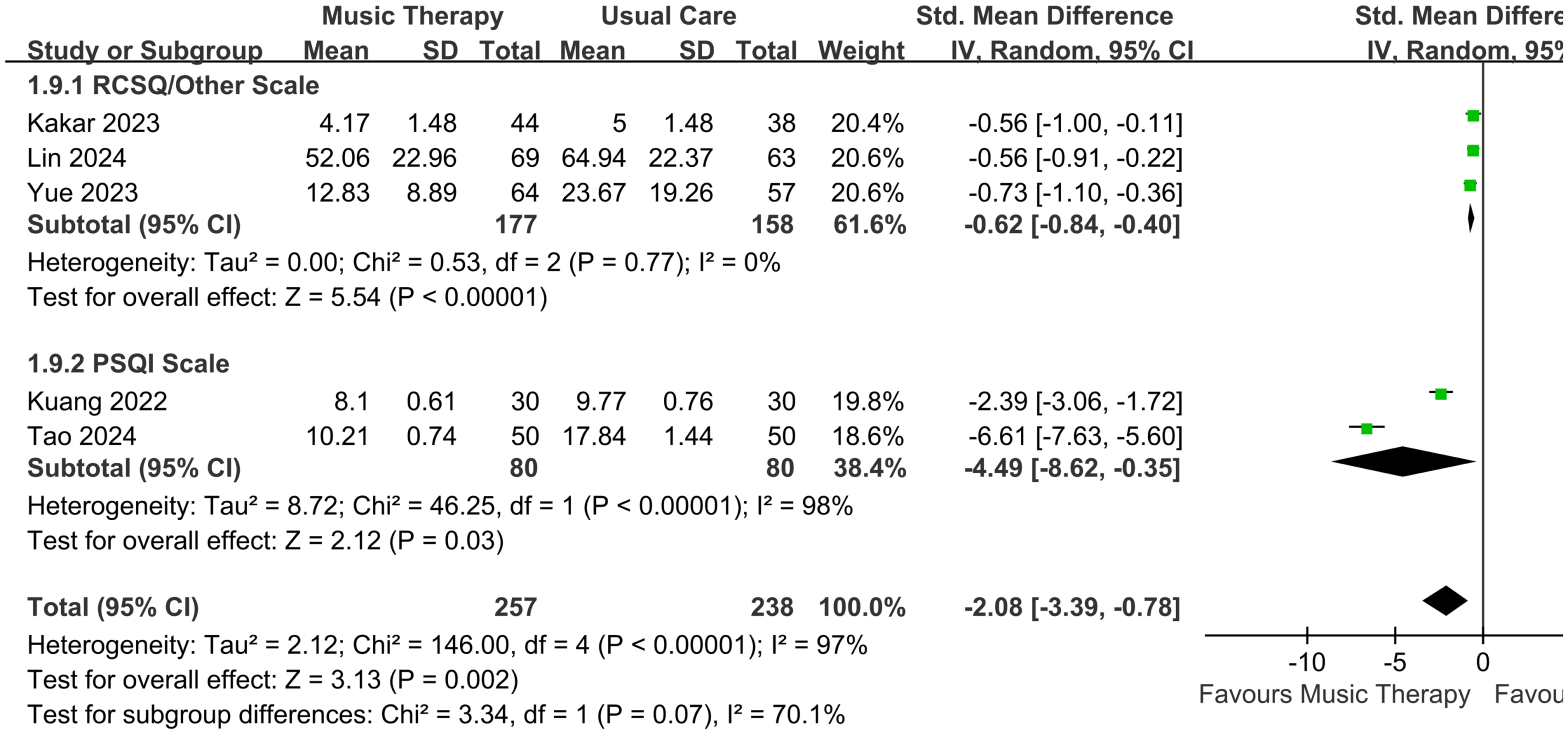
Forest plot of subgroup analysis for sleep quality based on different assessment scales (RCSQ vs. PSQI).

**Figure 9:**
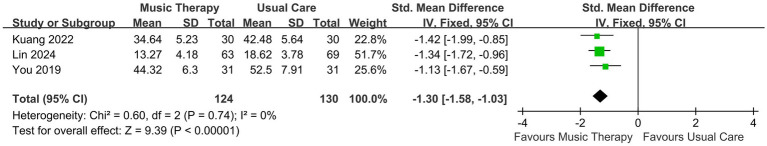
Forest plot for depression.

Subgroup analyses revealed significant associations between exercise type and psychological outcomes. For anxiety, sensitivity analysis excluding studies with methodological variations (14, 22) demonstrated a significant intervention effect (*SMD* = −0.79; *p* = 0.02). Regional subgroup analysis explained the residual heterogeneity (*I*^2^ for subgroup difference = 96.3%), showing a significant reduction in anxiety within the Chinese study cohort (*SMD* = −1.35; *p* < 0.001; *I*^2^ = 0%) compared with international studies (Figure 7). Regarding sleep quality, subgroup analysis based on assessment scales identified the Richards-Campbell Sleep Questionnaire (RCSQ) as a more consistent measure, with zero heterogeneity and significant improvements in acute sleep architecture (*SMD* = −0.62; *p* < 0.001; *I^2^* = 0%) (Figure 8). Conversely, substantial heterogeneity was observed in studies using the Pittsburgh Sleep Quality Index (*I^2^* = 98%). Depression levels were also significantly reduced (*SMD* = −1.30; *p* < 0.001) (Figure 9), while no significant difference was found in pain scores.

### 3.6 Certainty of evidence (GRADE)

Based on the GRADE system, the certainty of evidence for delirium incidence and ICU length of stay was rated as High, as these results were robust against risk of bias and inconsistency. Moderate certainty was assigned to depression and total hospital stay, downgraded primarily for performance bias or imprecision. Low to Very Low certainty was observed for mechanical ventilation, anxiety, and mortality due to substantial heterogeneity, wide confidence intervals, and small event rates. Detailed information regarding the GRADE certainty of evidence assessment and specific reasons for downgrading are provided in Supplementary Table S1.

## 4 Discussion

### 4.1 Association of music therapy with ICU delirium incidence

The primary objective of this systematic review and meta-analysis was to determine the efficacy of music therapy in reducing the incidence of delirium among adult ICU patients. Our findings indicate that music intervention is associated with a 51% reduction in the risk of developing delirium (RR = 0.49; 95% CI: 0.40 to 0.60). Of the 15 included studies, the vast majority of independent data from randomized controlled trials (15, 18, 21–25) strongly supported this conclusion. This meta-analysis aligns closely with the current international consensus in critical care medicine regarding non-pharmacological interventions for the prevention of ICU delirium (26). Clinical practice guidelines also explicitly recommend improving patients’ sensory environment and comfort to prevent and control acute brain dysfunction (1, 27). However, it is noteworthy that the multicenter randomized trials (16) conducted did not observe significant preventative effects. The underlying reasons for this include higher APACHE II scores in specific patient cohorts of these studies, more severe baseline conditions, and potentially later intervention timing. Notably, this reduction in the risk of acute brain dysfunction remained consistent after excluding clinical outliers in sensitivity analyses. Unlike previous meta-analyses that primarily focused on patient-reported pain or anxiety, this study prioritizes delirium, a clinical manifestation of acute cerebral impairment, as the primary endpoint. These results suggest that music therapy facilitates a shift in critical care management from providing simple psychological comfort toward implementing standardized non-pharmacological protocols for preventing brain dysfunction.

### 4.2 Pathophysiological pathways and improved sleep architecture

The reduction in delirium incidence can be explained by the modulation of neurobiological pathways. Critical illness and the ICU environment trigger neuroinflammatory responses and hyperactivation of the hypothalamic–pituitary–adrenal (HPA) axis, which are core components of the delirium pathophysiology. Auditory stimulation via music may facilitate the release of endorphins and dopamine, potentially counteracting the stress-induced neurotransmitter imbalance (28, 29). A critical factor identified in our analysis is the improvement of acute sleep architecture. Subgroup analysis confirmed that studies (16, 23, 24) using the Richars-Campbell Sleep Questionnaire (RCSQ) reported significantly improved sleep quality, with no heterogeneity (*I^2^* = 0%). In the ICU, restored sleep quality is essential for maintaining autonomic nervous system stability. By improving sleep, music therapy reduces the occurrence of psychomotor agitation and the subsequent requirement for sedative medications like benzodiazepines, which are known to increase delirium risk (30). This reduction in pharmacological dependency likely interrupts the iatrogenic cycle that contributes to cognitive impairment, thereby explaining the observed acceleration in weaning from mechanical ventilation and the shortening of ICU length of stay (19, 20, 25, 31).

### 4.3 Regional disparities and the influence of visiting policies

The regional subgroup analysis observed a significantly more pronounced effect in Chinese cohorts (19, 24, 31–33) (*SMD* = −1.35) for anxiety relief compared with international studies. This disparity may be attributed to fundamental differences in ICU structural characteristics and visiting policies (34). Many international centers have adopted open-visiting models that are recognized as effective protective factors against ICU-related stress. In contrast, most Chinese ICUs continue to maintain stringent “no-visitor” protocols, which subject patients to extreme emotional and sensory isolation. In such environments, music therapy serves as a critical compensatory source of sensory support and psychological comfort. Furthermore, the implementation of culturally congruent interventions, specifically traditional Chinese Five-Element music, plays a pivotal role in these results (19, 23, 24). By utilizing the distinct tonal characteristics of Gong, Shang, Jue, Zhi, and Yu, this modality aligns with the cultural esthetic and biological rhythms of Chinese patients. This alignment facilitates a deeper emotional resonance and a profound sense of safety, effectively mitigating the sympathetic overactivity induced by the sterile ICU environment. These findings underscore the critical value of personalized, culturally sensitive non-pharmacological care in optimizing outcomes for diverse patient populations.

### 4.4 Analysis of clinical confounders and sources of heterogeneity

To ensure the findings of this study are as accurate and interpretable as possible, it is necessary to examine several key ICU confounders, namely music type, intervention duration, cultural context, the physical environment, and concurrent sedation protocols.

First, the type of music and the intervention duration varied markedly across the included studies. While some trials utilized standardized classical or soothing music, others allowed patient-preferred selections (e.g., personalized playlists or slow-tempo tracks), which may optimize emotional resonance and enhance therapeutic efficacy. Moreover, sessions lasted 20 to 90 min and were administered 1 to 3 times daily. These wide differences in music selection and session length make it difficult to establish a single, uniform protocol for ICU clinicians. This lack of standardization also explains why the clinical outcomes differ across individual studies.

Second, cultural contexts and the physical ICU environment act as powerful external confounders. For instance, while many international centers utilize open-visiting models to alleviate patient stress, strict ‘no-visitor’ policies remain prevalent in Chinese ICUs. Consequently, music therapy provides essential emotional support for completely isolated patients, whereas it serves merely as an extra source of comfort in open units. In addition to these policy differences, the physical environment of the ICU, such as high ambient noise and frequent nursing disruptions, can directly lessen the calming effects of music. These variations in visiting policies and ward conditions create distinct clinical settings, making it difficult to compare the true effects of the intervention across studies.

Finally, concurrent sedation protocols are a critical factor that is often under-reported in intensive care studies. The specific choice and depth of sedation directly alter a patient’s neurological state. For example, dexmedetomidine inherently reduces delirium risk, whereas propofol and benzodiazepines are known to worsen cognitive impairment. Because music therapy serves as an adjunctive strategy, its true clinical effects can easily be masked or distorted by these underlying drug regimens. Therefore, future trials need to carefully control for and report sedation practices to make the evidence more reliable.

In summary, variations in intervention protocols, ward environments, and baseline sedation regimens constitute interconnected clinical confounders that complicate the direct evaluation of music therapy’s true efficacy across studies. Therefore, future trials might benefit from considering more consistent approaches to music delivery, accounting for environmental noise interference, and further exploring how concurrent sedation regimens interact with the intervention to better understand music therapy’s independent value.

### 4.5 Healthcare resource utilization and survival outcomes

Regarding the lack of a significant association between music therapy and short-term mortality (*p* = 0.89), these findings align with the clinical profile of music as a non-invasive, sensory-based intervention (22, 31, 32). Unlike pharmacological agents that provide immediate cardiovascular or respiratory support, music therapy does not directly address the acute physiological failures, such as hemodynamic collapse or multi-organ dysfunction, that are the primary drivers of ICU mortality. Consequently, music therapy should be viewed as an essential adjunctive tool for optimizing the quality of recovery rather than a primary resuscitative intervention for life-threatening conditions.

### 4.6 Methodological considerations and quality of evidence

The internal validity of our findings is supported by a rigorous assessment of the methodological quality of the 15 included trials. While 26.7% of the studies demonstrated a low overall risk of bias, the majority (53.3%) were categorized as having “some concerns,” and 20.0% were at high risk. A primary methodological challenge identified was the lack of blinding of participants and personnel (Domain D2) and outcome assessors (Domain D4). Due to the nature of music therapy, achieving true double-blinding is practically difficult in a clinical ICU setting. This inherent limitation often results in performance and detection bias. However, it is noteworthy that for our primary outcome—the incidence of delirium—the risk was partially mitigated by the use of standardized and validated clinical tools such as the CAM-ICU or CAM-ICU-7. These structured assessment scales provide a degree of objectivity that enhances the reliability of the results despite the lack of blinding.

Furthermore, our analysis revealed that reporting bias (Domain D5) remains a concern in half of the included studies due to the absence of accessible, pre-registered clinical trial protocols. This underscores the need for future critical care research to prioritize prospective registration and the publication of detailed statistical analysis plans to ensure transparency. Despite these methodological variations, the GRADE certainty of evidence for our primary outcome (delirium incidence) and ICU length of stay remained high. This high certainty suggests that the observed 51% reduction in delirium risk is robust and unlikely to be substantially altered by future studies. Conversely, the lower certainty assigned to outcomes like mechanical ventilation and anxiety reflects the high heterogeneity and imprecision observed in those specific data clusters.

## 5 Limitations

This study has several limitations. First, given the nature of the intervention, blinding clinicians and patients was infeasible, introducing potential performance bias. Second, substantial heterogeneity remained in exploratory outcomes such as anxiety and pain. Third, while the findings for delirium incidence and ICU length of stay are supported by high-quality evidence, the certainty of other outcomes is constrained by wide confidence intervals and a limited total number of observed clinical events. Finally, we were unable to control for all environmental confounders, including varying levels of background noise or specific sedation protocols, which may influence delirium outcomes.

## 6 Conclusion

This systematic review and meta-analysis of 15 RCTs indicate that music therapy is a safe and low-cost non-pharmacological intervention that significantly reduces the incidence of delirium in adult ICU patients. By effectively restoring acute sleep architecture and alleviating psychological distress, music therapy facilitates earlier weaning from mechanical ventilation and shortens ICU length of stay. While no direct impact on short-term mortality was observed, the clinical value of music therapy lies in its ability to optimize recovery trajectories and reduce healthcare resource utilization. Nevertheless, given the substantial heterogeneity and low certainty of evidence for several exploratory outcomes, clinical practitioners should interpret these findings with caution. Rather than an immediate blanket recommendation for routine integration into standard ICU and ERAS pathways, personalized application based on patient preference is advised until higher-certainty evidence becomes available.

## Data Availability

The original contributions presented in the study are included in the article/Supplementary material, further inquiries can be directed to the corresponding author.
