# Supplementary material for: Effects of music therapy on delirium, clinical outcomes, and psychological and sleep outcomes in adult ICU patients: a systematic review and meta-analysis
Source: Front Med (Lausanne). 2026 Jul 1;13:1857001. doi: 10.3389/fmed.2026.1857001 (PMC13369453; doi:10.3389/fmed.2026.1857001)
Supplement: Supplementary file 1 [file Supplementary_file_1.docx]

**Supplementary Material 1: Detailed Search Strategies for All Databases**

**Title of the Manuscript**: Effects of Music Therapy on Delirium, Clinical Outcomes, and Psychological and Sleep Outcomes in Adult ICU Patients: A Systematic Review and Meta-Analysis

**Search Date**: December 20, 2025 (All databases searched from inception to December 20, 2025)

**Total Records Identified**: 863

**----------------------------------------------------------------------**

1. Medline (via Ovid)

- Total Results: 152

[Delirium Domain]

- Line 1-13: Delirium/ OR Emergence Delirium/ OR delirium.mp. OR delirium of mixed origin.mp. OR mixed origin deliriums.mp. OR subacute delirium.mp. OR delirium, subacute.mp. OR subacute deliriums.mp. OR emergence delirium.mp. OR delirium, emergence.mp. OR agitated emergence.mp. OR emergence, agitated.mp. OR emergence agitation.mp. (Results: 29,128)

- Line 14-26: (Lines repeated in Ovid interface) Delirium/ OR Emergence Delirium/ OR delirium.mp. OR delirium of mixed origin.mp. OR mixed origin deliriums.mp. OR subacute delirium.mp. OR delirium, subacute.mp. OR subacute deliriums.mp. OR emergence delirium.mp. OR delirium, emergence.mp. OR agitated emergence.mp. OR emergence, agitated.mp. OR emergence agitation.mp. (Results: 29,128)

- Line 27-43: agitation, emergence.mp. OR agitations, emergence.mp. OR emergence excitement.mp. OR excitement, emergence.mp. OR postanesthetic excitement.mp. OR excitement, postanesthetic.mp. OR anesthesia delirium, anesthesia emergence.mp. OR emergence delirium, anesthesia.mp. OR postoperative delirium.mp. OR delirium, postoperative.mp. OR post-operative delirium.mp. OR delirium, post-operative.mp. OR post operative delirium.mp. OR acute delirium.mp. OR chronic delirium.mp. OR delier.mp. OR delire.mp.

- Line 44-58: deliria.mp. OR delirious manifestation.mp. OR delirious state.mp. OR delirious syndrome.mp. OR delirium acutum.mp. OR delirium after surgery.mp. OR delirium after surgical intervention.mp. OR delirium after surgical repair.mp. OR delirium after surgical resection.mp. OR Post-operative confusion.mp. OR post-operative deliria.mp. OR post-operative delirium.mp. OR postoperative confusion.mp. OR postoperative deliria.mp. OR postoperative delirious state.mp.

- Line 59-79: 25-item Delirium Observation Screening Scale.mp. OR delirium assessment instrument.mp. OR delirium assessment tool.mp. OR delirium evaluation.mp. OR delirium evaluation tool.mp. OR DOSS.mp. OR Delirium Observation Screening Scale.mp. OR DOS.mp. OR Delirium Observation Screening.mp. OR Delirium Observation Screening scale.mp. OR Delirium Observational Screening Scale.mp. OR DRS.mp. OR Delirium Rating Scale.mp. OR Trzepacz Delirium Rating Scale.mp. OR Trzepacz Delirium Symptom Rating Scale.mp. OR DRS-R98.mp. OR Delirium Rating Scale-98.mp. OR Delirium Rating Scale-98 revised.mp. OR Delirium Rating Scale-98-R.mp. OR Delirium Rating Scale-revised-98.mp. OR DRS-R-98.mp.

- Line 80-101: DRS-R-98 scale.mp. OR hyper-active delirium.mp. OR hypo-active delirium.mp. OR delirium screening scale in the intensive care unit.mp. OR ICDSC delirium checklist.mp. OR ICDSC questionnaire.mp. OR ICDSC scale.mp. OR ICDSC.mp. OR ICU delirium screen checklist.mp. OR ICU Delirium Screening Checklist.mp. OR Intensive Care Delirium Checklist.mp. OR Intensive Care Delirium Screening Checklist.mp. OR Intensive Care Unit Delirium Screening.mp. OR Intensive Care Unit Delirium Screening Checklist.mp. OR MDAS.mp. OR Memorial Delirium Assessment Scale.mp. OR NuDESC.mp. OR Nurse Delirium Screening Scale.mp. OR Nursing Delirium Assessment Screen.mp. OR Nursing Delirium Scale.mp. OR Nursing Delirium Screen.mp. OR Nursing Delirium Screening Scale.mp.

- Line 102 [Combined Delirium Index]: 1 OR 2 OR ... OR 101 (Total Results: 75,519)

[Music Domain]

- Line 103-113: Music Therapy/ OR Acoustic Stimulation/ OR Voice Training/ OR Voice Quality/ OR Voice/ OR Sound/ OR Auditory Perception/ OR music.mp. OR songs.mp. OR song.mp. OR vocal melody.mp.

- Line 114-138: Music/ OR melodies, vocal.mp. OR melody, vocal.mp. OR vocal melodies.mp. OR classical music.mp. OR jazz music.mp. OR rap music.mp. OR hip hop music.mp. OR music, hip hop.mp. OR music therapy.mp. OR therapy, music.mp. OR acoustic stimulation.mp. OR stimulation, auditory.mp. OR stimulation, acoustic.mp. OR auditory stimulation.mp. OR voice.mp. OR Voices.mp. OR voice quality.mp. OR qualities, voice.mp. OR quality, voice.mp. OR voice qualities.mp. OR voice training.mp. OR trainings, voice.mp. OR training, voice.mp. OR voice trainings.mp.

- Line 139-160: sound.mp. OR sounds.mp. OR sonic radiation.mp. OR radiation, sonic.mp. OR radiations, sonic.mp. OR sonic radiations.mp. OR sound waves.mp. OR sound wave.mp. OR wave, waves, acoustic waves.mp. OR acoustic wave.mp. OR wave, acoustic.mp. OR waves, acoustic.mp. OR elastic waves.mp. OR elastic wave.mp. OR wave, elastic.mp. OR waves, elastic.mp. OR auditory perception.mp. OR Auditory Perception/ OR perception, auditory.mp. OR auditory processing.mp. OR processing, auditory.mp. OR melotherapy.mp. OR therapy, music.mp.

- Line 161-180: acoustic stimulus.mp. OR audio stimulus.mp. OR auditory stimulus.mp. OR sound stimulation.mp. OR stimulation, sound.mp. OR laryngeal voice.mp. OR vox.mp. OR complex sound.mp. OR sound propagation.mp. OR audition.mp. OR auditory function.mp. OR hearing conservation.mp. OR noise perception.mp. OR phantom hearing.mp. OR sound perception.mp. OR five-tone.mp. OR five-tone music.mp. OR five-tone music therapy.mp. OR five-element music.mp. OR five-element music therapy.mp.

- Line 181 [Combined Music Index]: 103 OR 104 OR ... OR 180 (Total Results: 303,231)

[Intensive Care Domain]

- Line 182-220: Intensive Care Units/ OR Critical Care/ OR Critical Illness/ OR Burn Units/ OR Coronary Care Units/ OR Intensive Care Units, Pediatric/ OR Recovery Room/ OR Respiratory Care Units/ OR intensive care units.mp. OR intensive care unit.mp. OR unit, intensive care.mp. OR icu intensive care units.mp. OR critical care.mp. OR care, critical.mp. OR intensive care.mp. OR care, intensive.mp. OR surgical intensive care.mp. OR care, surgical intensive.mp. OR intensive care, surgical.mp. OR critical illness.mp. OR critical illnesses.mp. OR illness, critical.mp. OR illnesses, critical.mp. OR critically ill.mp. OR critical care unit.mp. OR general ICU.mp. OR GICU.mp. OR GICUs.mp. OR ICU's.mp. OR intensive therapy unit.mp. OR intensive treatment unit.mp. OR medical-surgery ICU.mp. OR Medical surgical ICU.mp. OR Medical surgical ICUs.mp. OR Medico-surgical ICU.mp. OR Surgery medical ICU.mp. OR surgical-medical ICUs.mp. OR Surgical medical ICU.mp. OR intensive care, paediatric.mp.

- Line 221-256: intensive care, pediatric.mp. OR intensive therapy.mp. OR paediatric intensive care.mp. OR pediatric intensive care.mp. OR therapy, intensive.mp. OR burn center.mp. OR burn centers.mp. OR burn unit.mp. OR burn units.mp. OR center, burn.mp. OR centers, burn.mp. OR unit, burn.mp. OR units, burn.mp. OR coronary care unit.mp. OR coronary care units.mp. OR care unit, coronary.mp. OR care units, coronary.mp. OR unit, coronary care.mp. OR units, coronary care.mp. OR units, coronary care.mp. OR icu, pediatric.mp. OR icus, pediatric.mp. OR intensive care units, pediatric.mp. OR pediatric icu.mp. OR pediatric icus.mp. OR pediatric intensive care unit.mp. OR pediatric intensive care units.mp. OR hospital recovery room.mp. OR hospital recovery rooms.mp. OR recovery room.mp. OR recovery room, hospital.mp. OR recovery rooms.mp. OR recovery rooms, hospital.mp. OR room, hospital recovery.mp. OR room, recovery.mp. OR rooms, hospital recovery.mp.

- Line 257-299: rooms, recovery.mp. OR care unit, respiratory.mp. OR care units, respiratory.mp. OR respiratory care unit.mp. OR respiratory care units.mp. OR unit, respiratory care.mp. OR units, respiratory care.mp. OR early goal directed therapy.mp. OR early goal-directed therapies.mp. OR early goal-directed therapy.mp. OR care, neonatal intensive.mp. OR infant, newborn, intensive care.mp. OR intensive care, neonatal.mp. OR neonatal intensive care.mp. OR ICU.mp. OR General Intensive Care Unit.mp. OR MICU.mp. OR Medical Intensive Care Unit.mp. OR SICU.mp. OR Surgical Intensive Care Unit.mp. OR EICU.mp. OR Emergency Intensive Care Unit.mp. OR NICU.mp. OR Neonatal Intensive Care Unit.mp. OR PICU.mp. OR Pediatric Intensive Care Unit.mp. OR CCU.mp. OR Coronary Care Unit.mp. OR CSICU.mp. OR Cardiac Surgery Intensive Care Unit.mp. OR NSICU.mp. OR Neurosurgical Intensive Care Unit.mp. OR RICU.mp. OR Respiratory Intensive Care Unit.mp. OR UICU.mp. OR Urology Intensive Care Unit.mp. OR BICU.mp. OR Burn Intensive Care Unit.mp. OR AICU.mp. OR Anesthesia Intensive Care Unit.mp. OR TICU.mp. OR Transplant Intensive Care Unit.mp. OR OICU.mp. OR Obstetric Intensive Care Unit.mp.

- Line 300-329: Intensive Care Units, Neonatal/ OR intensive care units, neonatal.mp. OR newborn intensive care unit.mp. OR newborn intensive care units.mp. OR cardiac SICU.mp. OR cardiac surgery ICU.mp. OR cardiac surgical ICU.mp. OR cardiac surgical intensive care unit.mp. OR Cardio-surgery ICU.mp. OR Cardio-surgery intensive care unit.mp. OR Cardio-surgical ICU.mp. OR Cardio-surgical intensive care unit.mp. OR Cardio-thoracic intensive care unit.mp. OR cardiothoracic critical care unit.mp. OR cardiothoracic ICU.mp. OR cardiothoracic intensive care unit.mp. OR cardiothoracic surgery intensive care unit.mp. OR cardiothoracic surgical intensive care unit.mp. OR cardiovascular surgery intensive care unit.mp. OR cardiovascular surgical intensive care unit.mp. OR icu, neonatal.mp. OR icu, newborn.mp. OR icus, neonatal.mp. OR icus, newborn.mp. OR neonatal icu.mp. OR neonatal icus.mp. OR neonatal intensive care units.mp. OR heart surgery ICU.mp. OR newborn icu.mp. OR newborn icus.mp.

- Line 330 [Combined ICU Index]: 182 OR 183 OR ... OR 329 (Total Results: 411,881)

[Final Intersection]

- Line 331 [Final Boolean Restrict]: 102 AND 181 AND 330 (Total Results: 152)

----------------------------------------------------------------------

2. Embase (via Ovid)

- Total Results: 478

- Line 1-98: [Delirium-related terms including headings and text words] (delirium/ OR postoperative delirium/ OR delirium assessment/ OR Delirium Observation Screening/ OR Delirium Rating Scale/ OR hyperactive delirium/ OR hypoactive delirium/ OR Intensive Care Delirium Screening Checklist/ OR Memorial Delirium Assessment Scale/ OR Nursing Delirium Screening Scale/ OR comprehensive .mp. search strings for subacute delirium, emergence agitation, anesthetic excitement, DOSS, DOS, DRS, ICDSC, MDAS, NuDESC).

- Line 99 [Combined Delirium Index]: 1 OR 2 OR ... OR 98 (Total Results: 99,992)

- Line 100-176: [Music and Auditory stimulation terms] (music/ OR music therapy/ OR auditory stimulation/ OR voice training/ OR voice/ OR sound/ OR hearing/ OR comprehensive .mp. variations including acoustic stimulation, sound perception, traditional five-tone/five-element music therapies).

- Line 177 [Combined Music Index]: 100 OR 101 OR ... OR 176 (Total Results: 381,493)

- Line 178-321: [ICU and Critical care environment terms] (intensive care unit/ OR intensive care/ OR critical illness/ OR extensive text words for MICU, SICU, NICU, PICU, CCU, RICU, BICU, GICU, adult and pediatric clinical settings).

- Line 322 [Combined ICU Index]: 178 OR 179 OR ... OR 321 (Total Results: 784,228)

- Line 323 [Final Intersection]: 99 AND 177 AND 322 (Total Results: 478)

----------------------------------------------------------------------

3. Cochrane Library (via Ovid / Wiley)

- Total Results: 151

- Line 1-42: [Delirium Domain Keywords and MeSH Headings] (Acute brain dysfunction, emergence delirium, postoperative confusion, and standardized validation screening tools).

- Line 43 [Combined Delirium Index]: 1 OR 2 OR ... OR 42 (Total Results: 10,654)

- Line 44-114: [Auditory Intervention Domain Keywords and MeSH Headings] (Therapeutic acoustic stimulation, personalized songs, classical arrangements, and environmental sound interventions).

- Line 115 [Combined Music Index]: 44 OR 45 OR ... OR 114 (Total Results: 27,035)

- Line 116-240: [Intensive Care Setting Keywords and MeSH Headings] (Respiratory, surgical, medical, and coronary intensive care units, recovery environments, and critical illness states).

- Line 241 [Combined ICU Index]: 116 OR 117 OR ... OR 240 (Total Results: 57,977)

- Line 242 [Final Intersection]: 43 AND 115 AND 241 (Total Results: 151)

----------------------------------------------------------------------

4. Chinese Databases (CNKI, Wanfang, VIP, CBM)

4.1 China National Knowledge Infrastructure (CNKI)

- Search Field: Abstract/Title/Keywords (篇关摘)

- Formulation:

#1 ("音乐疗法" OR "音乐" OR "五行音乐" OR "旋律" OR "音乐治疗" OR "音频" OR "音乐干预" OR "声音疗法" OR "录音干预" OR "现场演奏" OR "白噪音" OR "自然声" OR "声刺激" OR "五音" OR "宫调" OR "商调" OR "角调" OR "羽调" OR "徵调")

#2 ("谵妄" OR "谵妄状态" OR "谵妄综合征" OR "DSM-5" OR "ICD-10" OR "CAM-ICU" OR "ICDSC")

#3 ("重症医学科" OR "重症医学" OR "重症监护室" OR "ICU" OR "SICU" OR "MICU" OR "PICU" OR "NICU" OR "CCU" OR "ECU" OR "EICU" OR "MCU" OR "TICU" OR "综合ICU" OR "专科ICU")

#4 Logic: #1 AND #2 AND #3

- Total Results: 33

4.2 VIP Database (CQVIP)

- Search Field: Title or Keywords (题名或关键词)

- Formulation: Same logic terms utilized as the CNKI search query (#1 AND #2 AND #3).

- Total Results: 2

4.3 Chinese Biomedical Literature Database (CBM)

- Search Field: Abstract (摘要)

- Formulation: Applied equivalent Mandarin components filtered within the abstract index (#1 AND #2 AND #3).

- Total Results: 12

4.4 Wanfang Data

- Search Field: Topic (主题)

- Formulation: Executed subject-heading query mapping across all music keywords, delirium diagnostics, and critical care units (#1 AND #2 AND #3).

- Total Results: 35
