# Supplementary material for: Effects of music therapy on delirium, clinical outcomes, and psychological and sleep outcomes in adult ICU patients: a systematic review and meta-analysis
Source: Front Med (Lausanne). 2026 Jul 1;13:1857001. doi: 10.3389/fmed.2026.1857001 (PMC13369453; doi:10.3389/fmed.2026.1857001)
Supplement: Supplementary file 2 [file Supplementary_file_2.pdf]

Certainty assessment							№ of patients		Effect		Certainty	Importance
№ of studies	Study design	Risk of bias	Inconsistency	Indirectness	Imprecision	Other considerations	Music Therapy	Usual Care	Relative (95% CI)	Absolute (95% CI)		
**Incidence of Delirium**												
11	randomised trials	not serious	not serious	not serious	not serious	none	132/584 (22.6%)	277/581 (47.7%)	**RR 0.49** (0.40 to 0.60)	**243 fewer per 1,000** (from 286 fewer to 191 fewer)	⊕⊕⊕⊕ High	CRITICAL
**Coma-free days**												
3	randomised trials	not serious	serious[a]	not serious	serious[b]	none	118	104	-	SMD **0.4 SD higher** (0.25 lower to 1.06 higher)	⊕⊕◯◯ Low[a,b]	CRITICAL
**Short-term Mortality**												
4	randomised trials	not serious	serious[c]	not serious	very serious[d]	none	36/208 (17.3%)	33/191 (17.3%)	**RR 1.05** (0.53 to 2.07)	**9 more per 1,000** (from 81 fewer to 185 more)	⊕◯◯◯ Very low[c,d]	CRITICAL
**ICU LOS**												
8	randomised trials	not serious	not serious	not serious	not serious	none	424	417	-	MD **1.07 lower** (1.8 lower to 0.33 lower)	⊕⊕⊕⊕ High	IMPORTANT
**Hospital LOS**												
3	randomised trials	not serious	not serious	not serious	serious[e]	none	146	134	-	MD **1.37 lower** (4.06 lower to 1.33 higher)	⊕⊕⊕◯ Moderate[e]	IMPORTANT
**Duration of mechanical ventilation**												
5	randomised trials	not serious	very serious[f]	not serious	serious[g]	none	283	273	-	SMD **0.64 SD lower** (1.29 lower to 0.02 higher)	⊕◯◯◯ Very low[f,g]	CRITICAL
**Anxiety**												
7	randomised trials	not serious	very serious[h]	not serious	serious[g]	none	214	201	-	SMD **0.9 SD lower** (1.83 lower to 0.04 higher)	⊕◯◯◯ Very low[g,h]	IMPORTANT
**Depression**												
3	randomised trials	serious[i]	not serious	not serious	not serious	none	124	130	-	SMD **1.3 SD lower** (1.58 lower to 1.03 lower)	⊕⊕⊕◯ Moderate[i]	IMPORTANT
**CPOT score**												
6	randomised trials	not serious	very serious[j]	not serious	serious[j]	none	256	232	-	SMD **0.56 SD lower** (1.29 lower to 0.17 higher)	⊕◯◯◯ Very low[j]	IMPORTANT
**Sleep Quality**												
5	randomised trials	not serious	very serious[k]	not serious	not serious	none	257	238	-	SMD **2.08 SD lower** (3.39 lower to 0.78 lower)	⊕⊕◯◯ Low[k]	IMPORTANT

CI: confidence interval; **MD:** mean difference; **RR:** risk ratio; **SMD:** standardised mean difference

Explanations

a. Downgraded by one level due to moderate statistical heterogeneity (I^2=55%).
b. Downgraded by one level as the 95% confidence interval crosses the line of no effect and the total sample size is small.
c. Downgraded by one level due to moderate statistical heterogeneity (I^2=46%) and conflicting results across studies.
d. Downgraded by two levels due to very wide 95% CI and small number of events.
e. Downgraded by one level as the 95% CI crosses the line of no effect and the sample size is small.
f. Downgraded by two levels due to very high statistical heterogeneity (I^2=93%) and instability of results in sensitivity analysis.
g. Downgraded by one level as the 95% CI crosses the line of no effect and the result is not statistically significant (P = 0.06).
h. Downgraded by two levels due to extremely high statistical heterogeneity (I^2=94%) and significant differences between subgroups (Chinese vs International studies).
i. Downgraded by one level due to lack of blinding (performance bias), which is inherent in music therapy interventions.
j. Downgraded by two levels for inconsistency (I^2=93%) and one level for imprecision (95% CI crosses the line of no effect).
k. Downgraded by two levels for very high heterogeneity (I^2=97%) primarily driven by different measurement scales.
