# Supplementary material for: Effects of music therapy on delirium, clinical outcomes, and psychological and sleep outcomes in adult ICU patients: a systematic review and meta-analysis
Source: Front Med (Lausanne). 2026 Jul 1;13:1857001. doi: 10.3389/fmed.2026.1857001 (PMC13369453; doi:10.3389/fmed.2026.1857001)

Supplementary Figure S1. Forest plot for the incidence of delirium (sensitivity analysis).


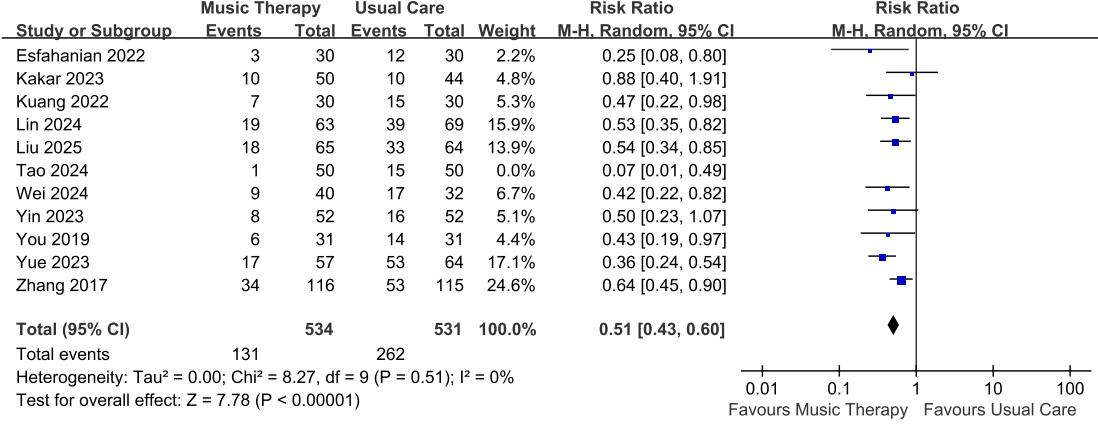


Supplementary Figure S2. Funnel plot for the incidence of delirium (sensitivity analysis).


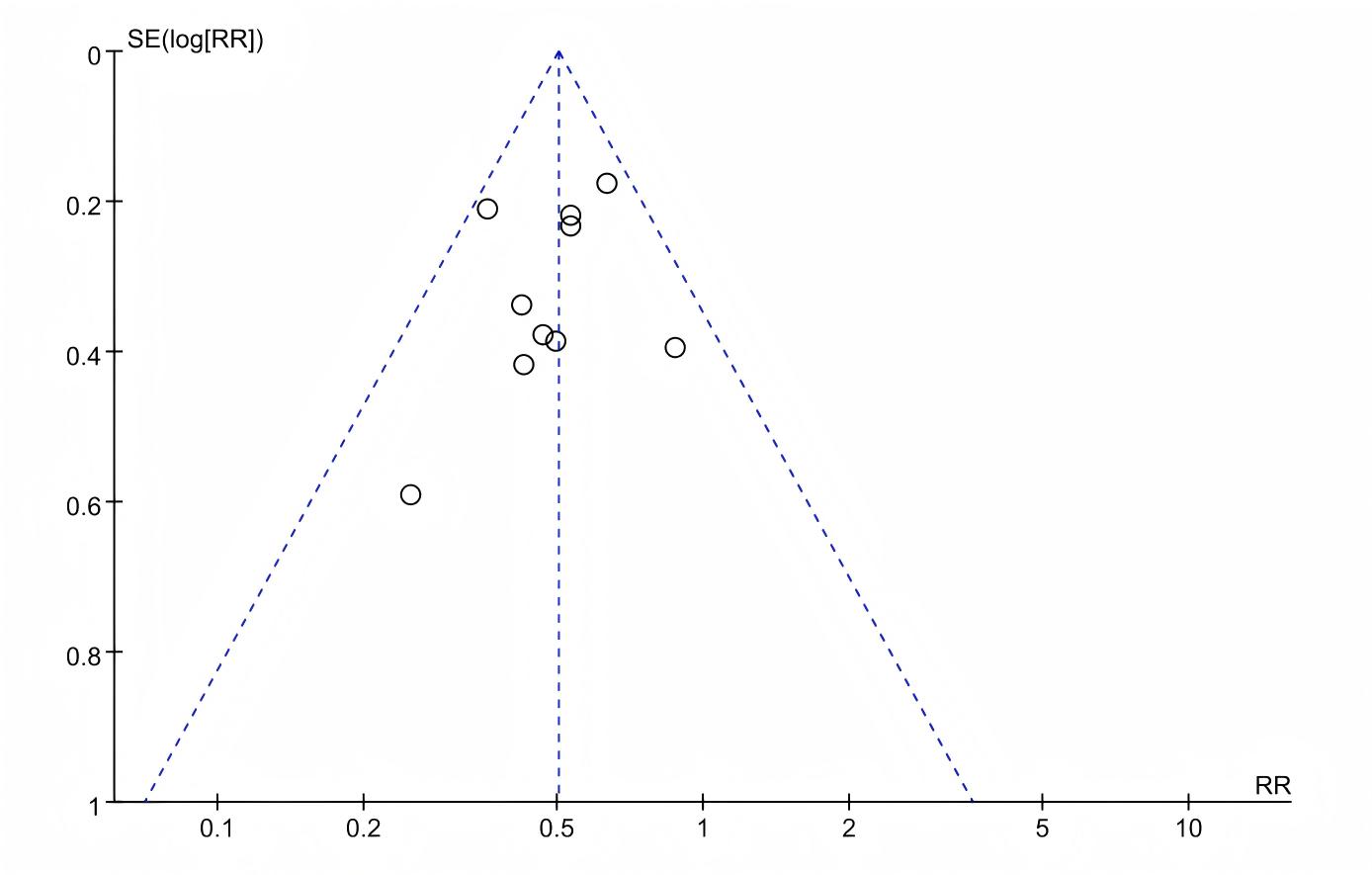


Supplementary Figure S3. Forest plot for coma-free days.


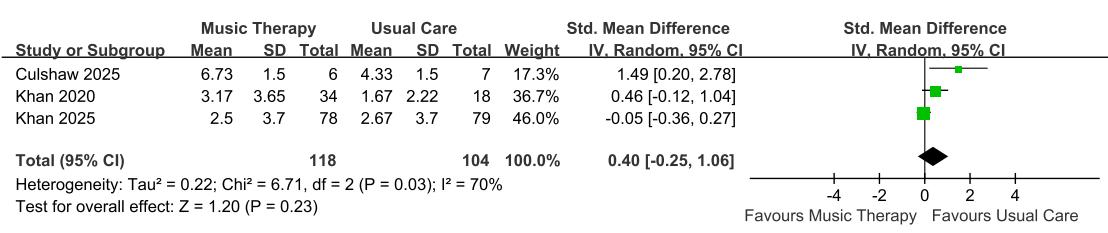


Supplementary Figure S4. Funnel plot for coma-free days.


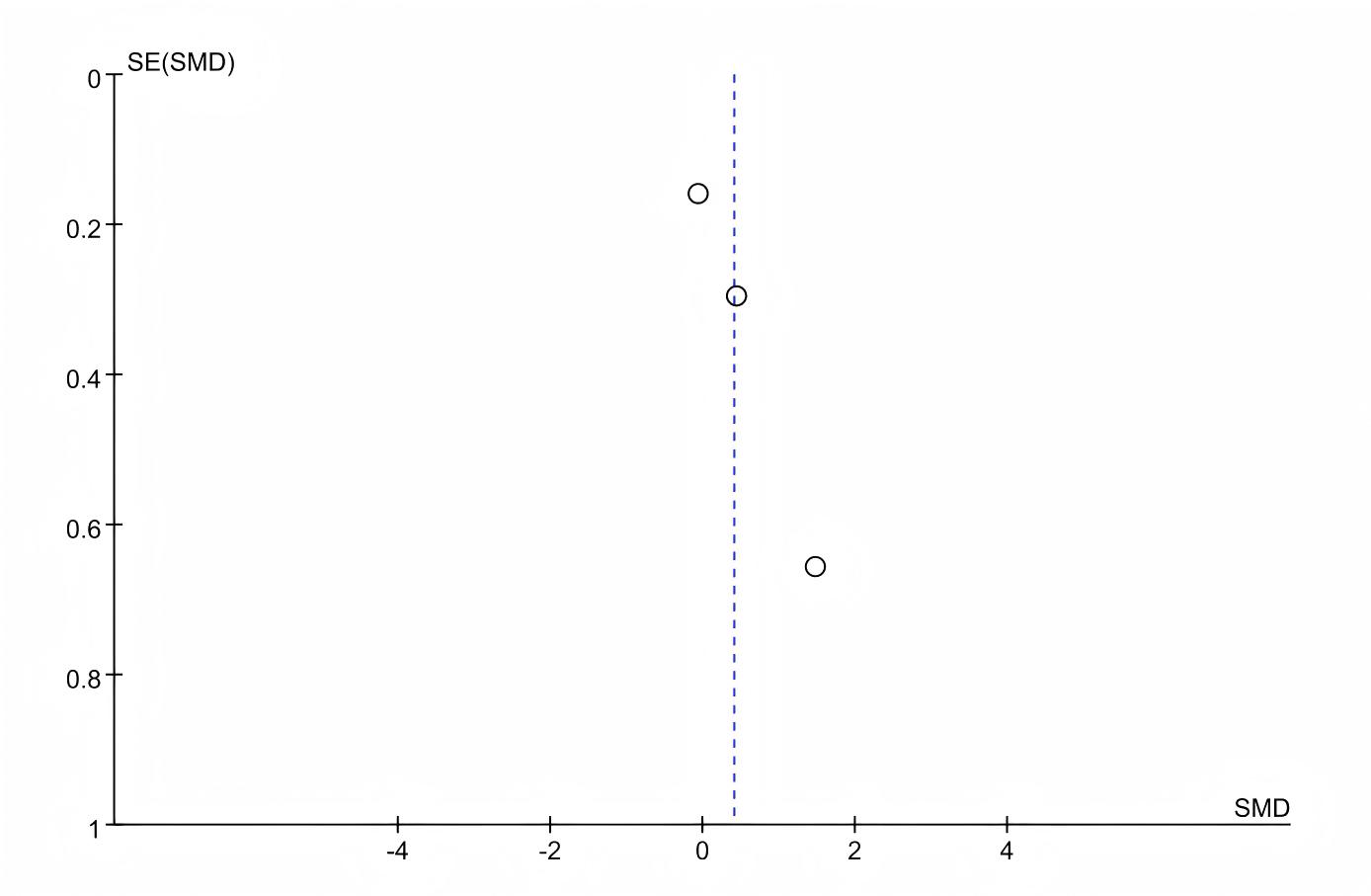


Supplementary Figure S5. Forest plot for coma-free days (sensitivity analysis).


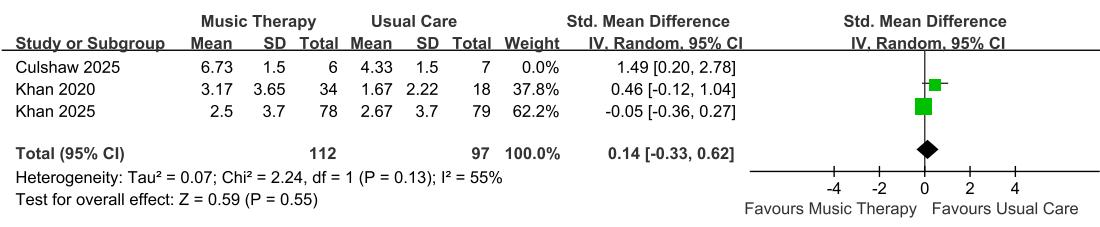


Supplementary Figure S6. Funnel plot for coma-free days (sensitivity analysis).


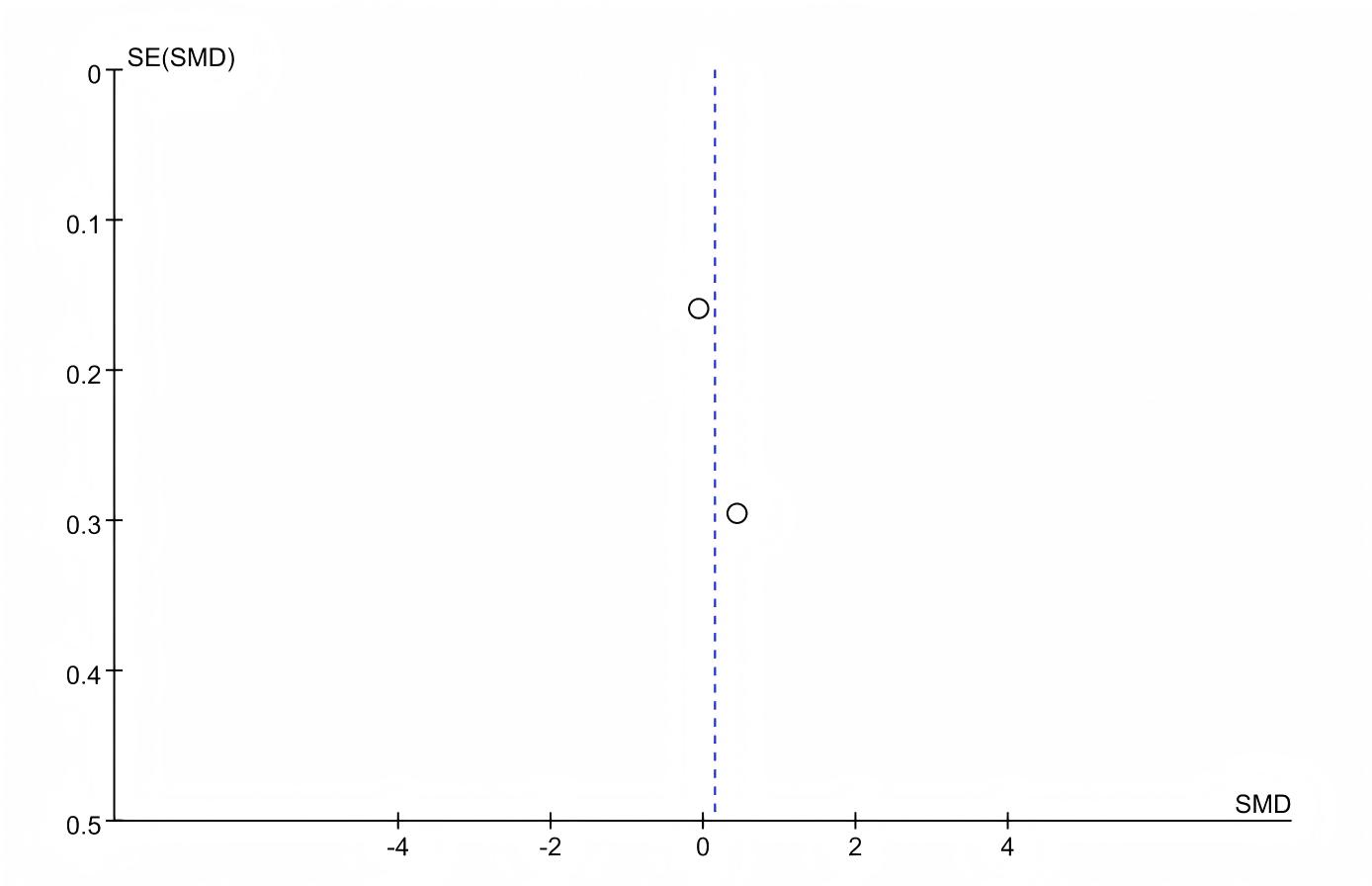


Supplementary Figure S7. Forest plot for short-term mortality.


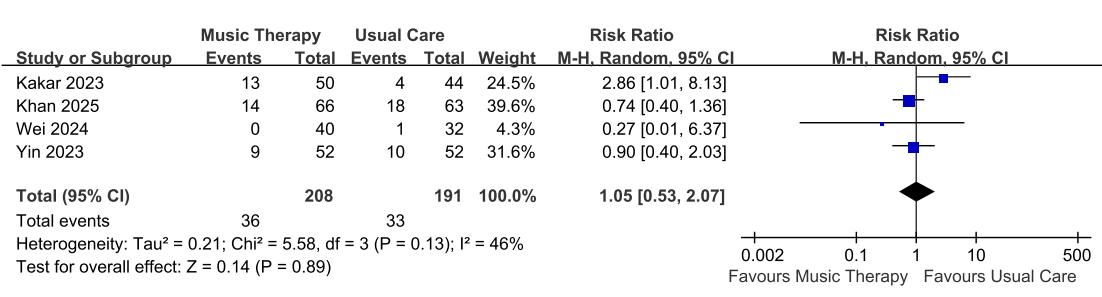


Supplementary Figure S8. Funnel plot for short-term mortality.


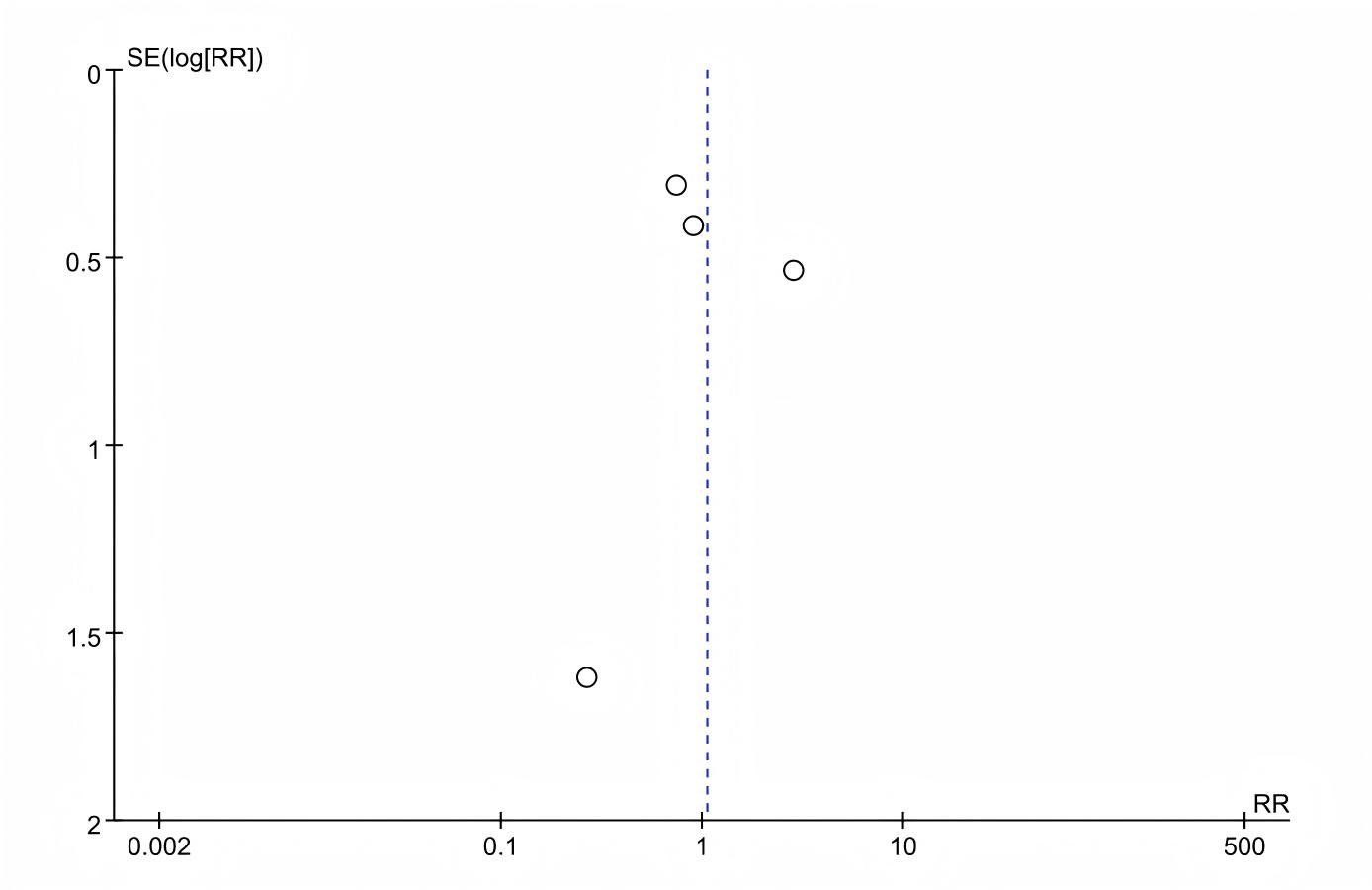


Supplementary Figure S9. Forest plot for duration of mechanical ventilation





Supplementary Figure S10. Funnel plot for duration of mechanical ventilation.


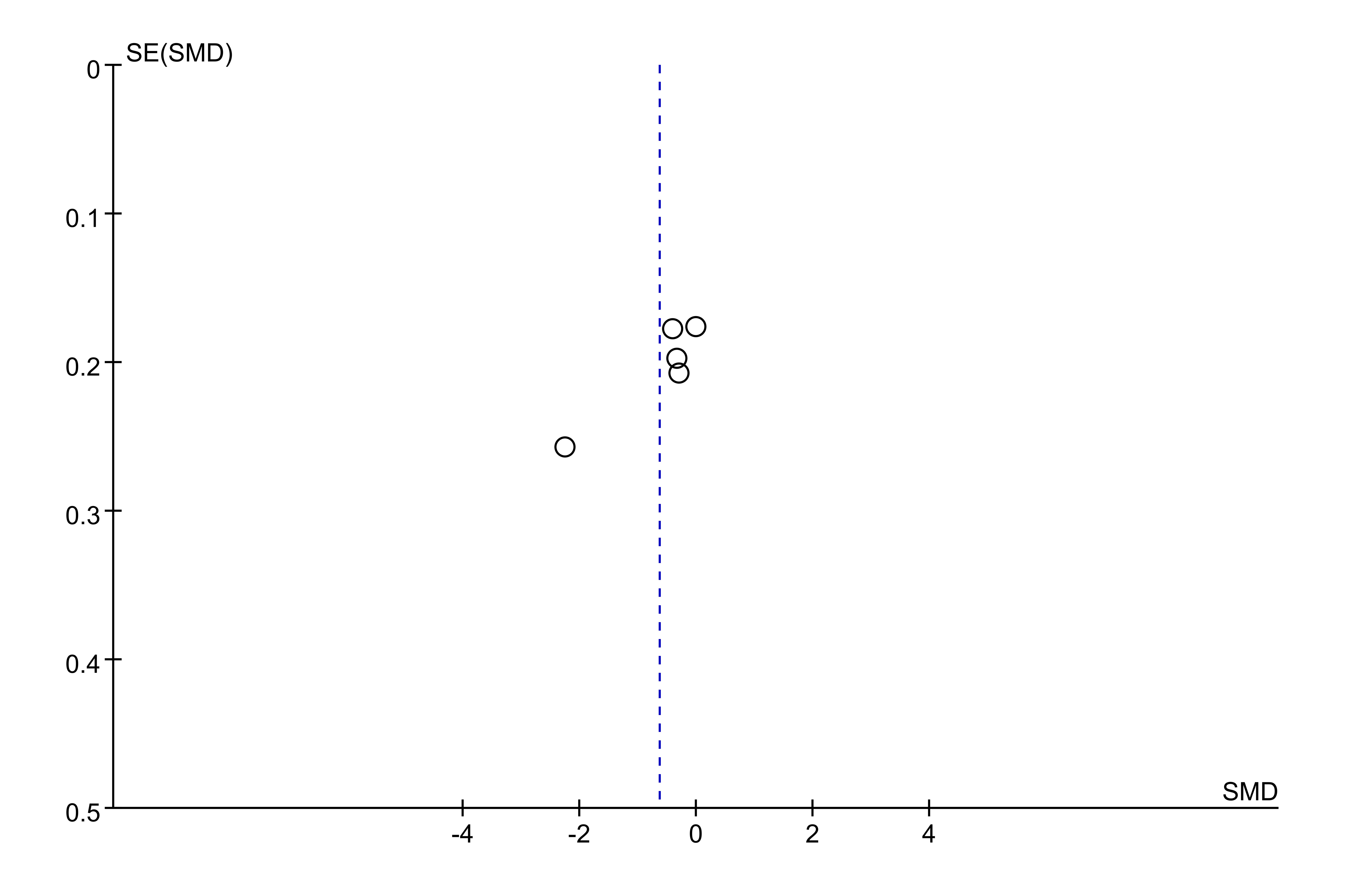


Supplementary Figure S11. Forest plot for ICU length of stay (LOS).





Supplementary Figure S12. Funnel plot for ICU length of stay (LOS).


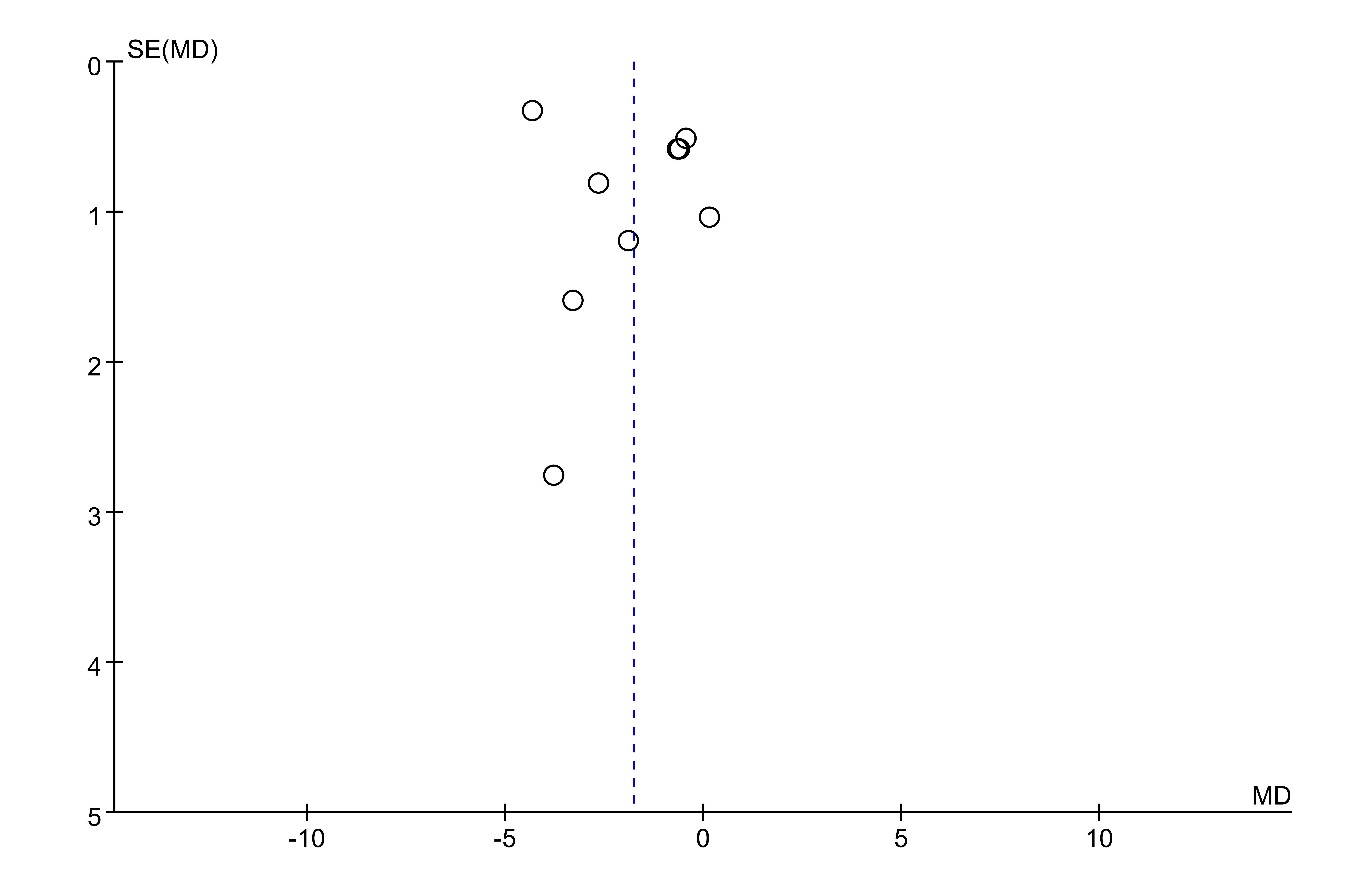


Supplementary Figure S13. Funnel plot for ICU length of stay (sensitivity analysis).


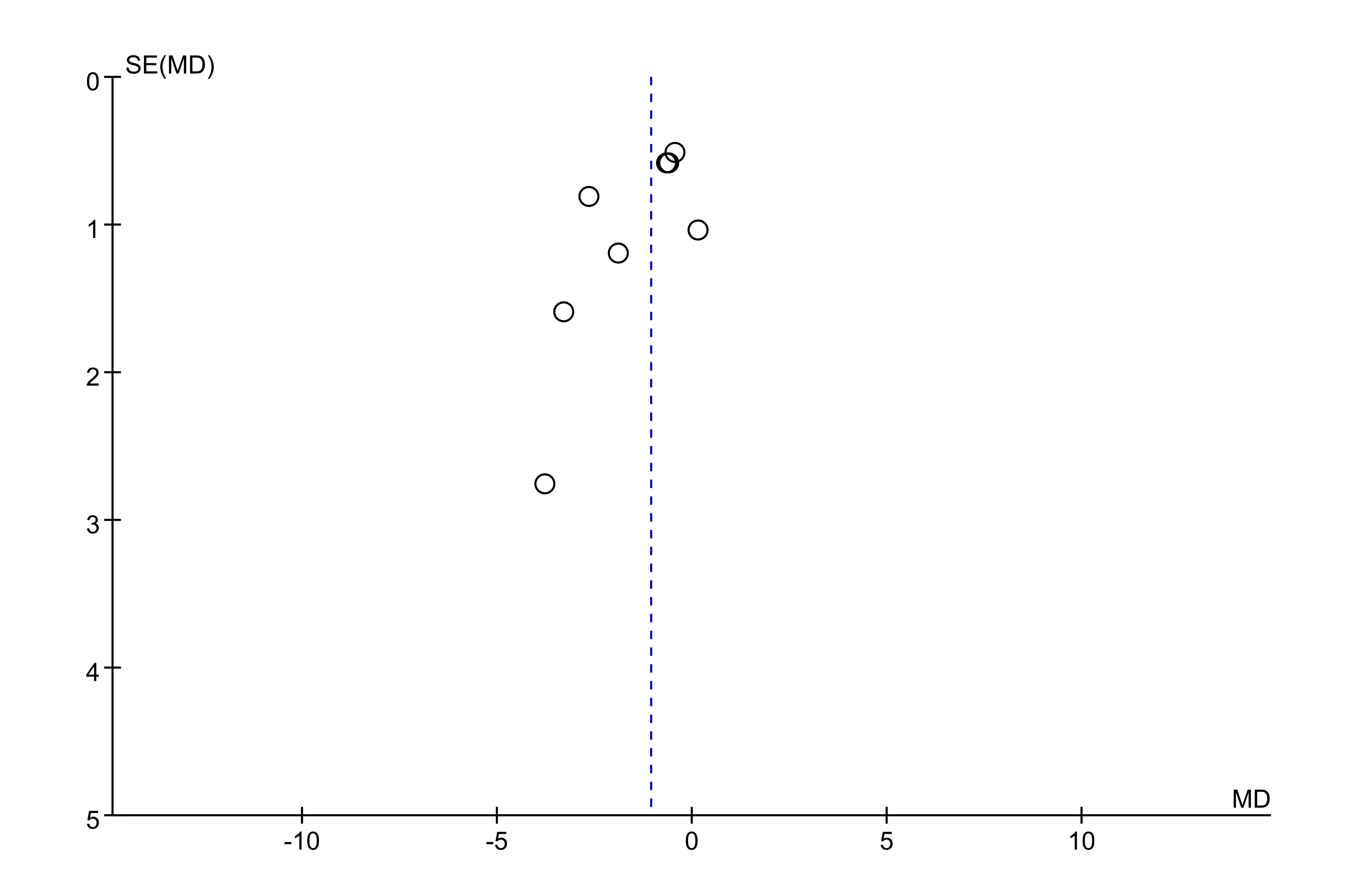


Supplementary Figure S14. Funnel plot for hospital length of stay (LOS).


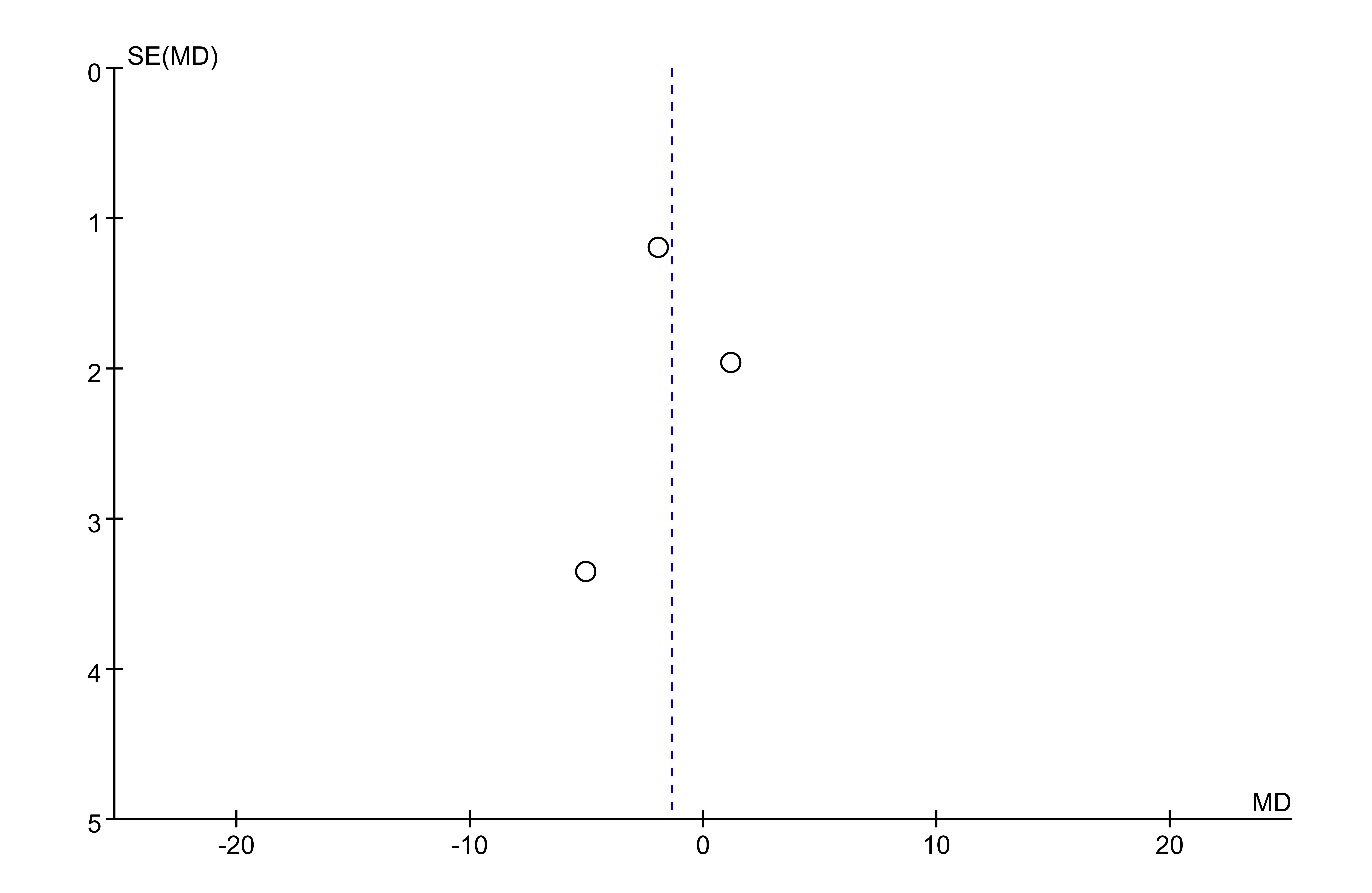


Supplementary Figure S15. Funnel plot for anxiety (sensitivity and subgroup analysis).


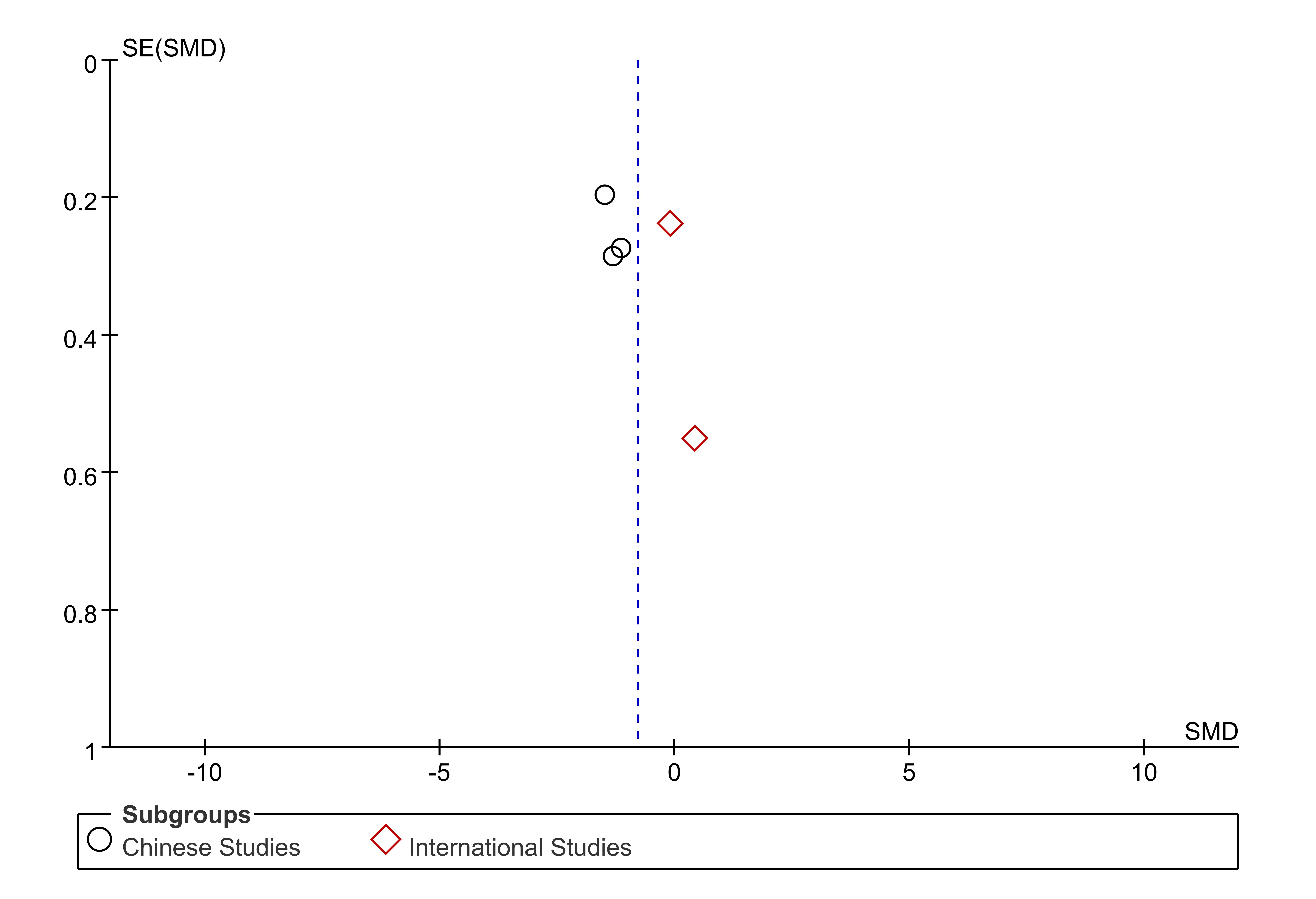


Supplementary Figure S16.Funnel plot for sleep quality (subgroup analysis).


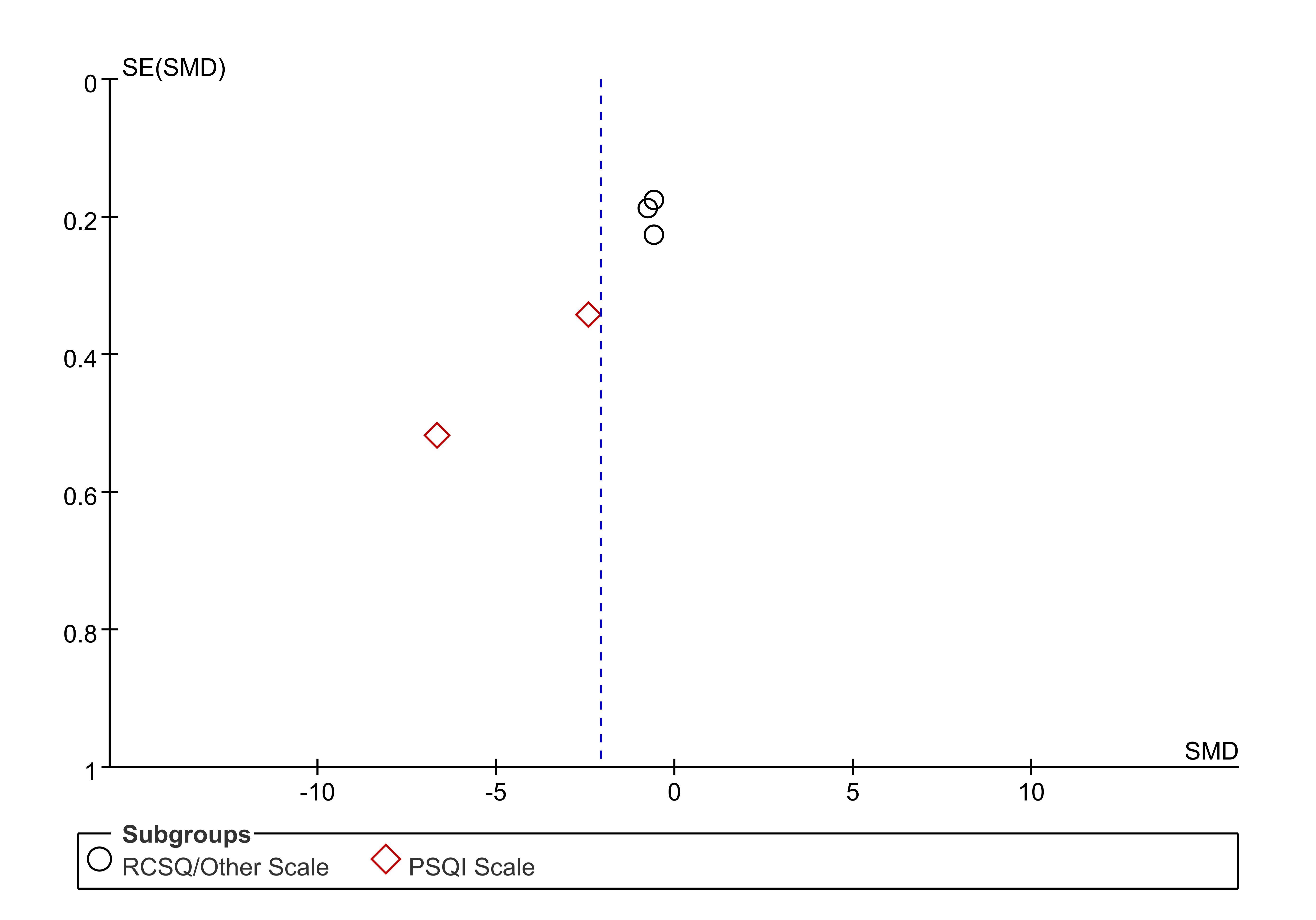


Supplementary Figure S17. Funnel plot for depression.


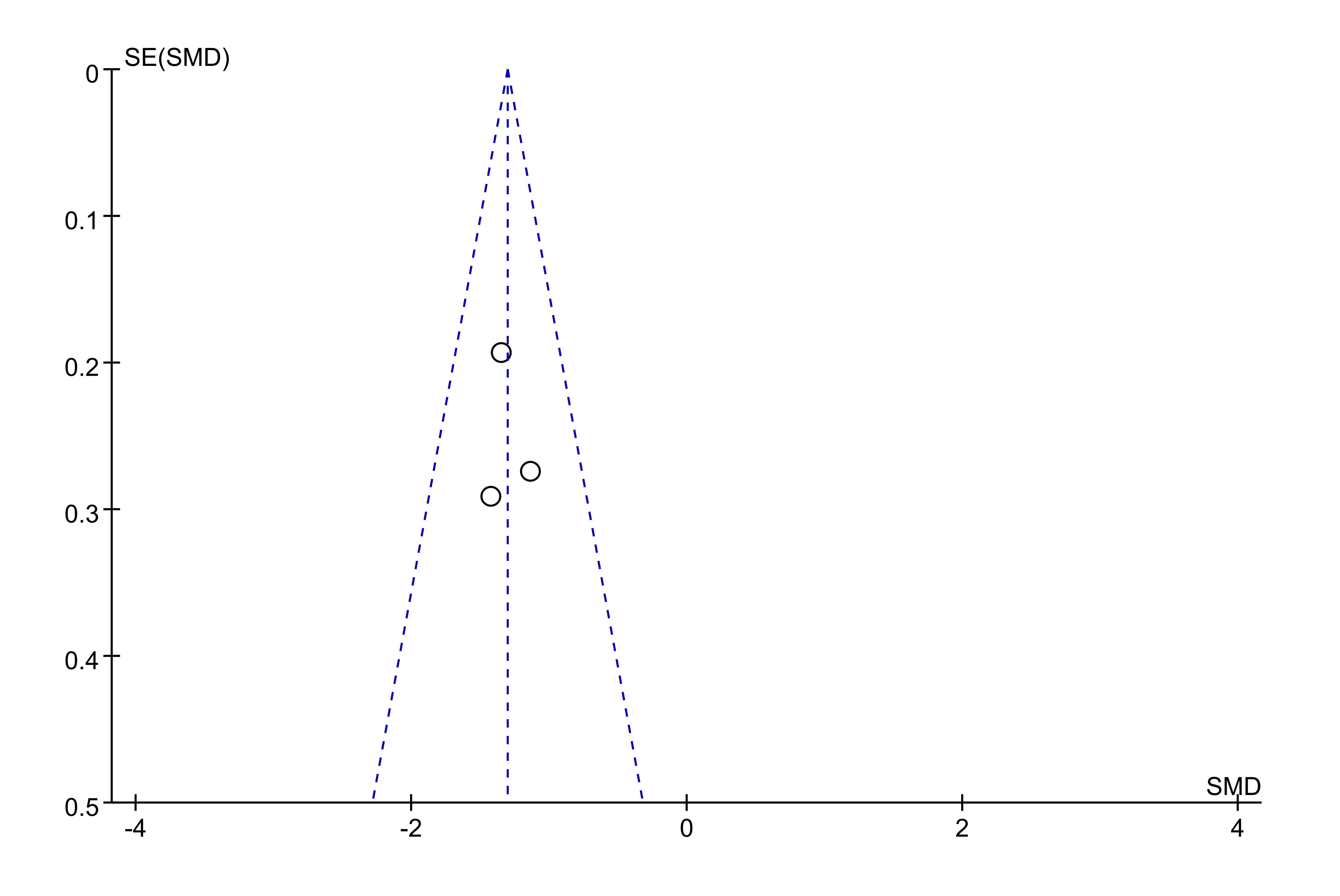


Supplementary Figure S18. Forest plot for CPOT (pain scores).


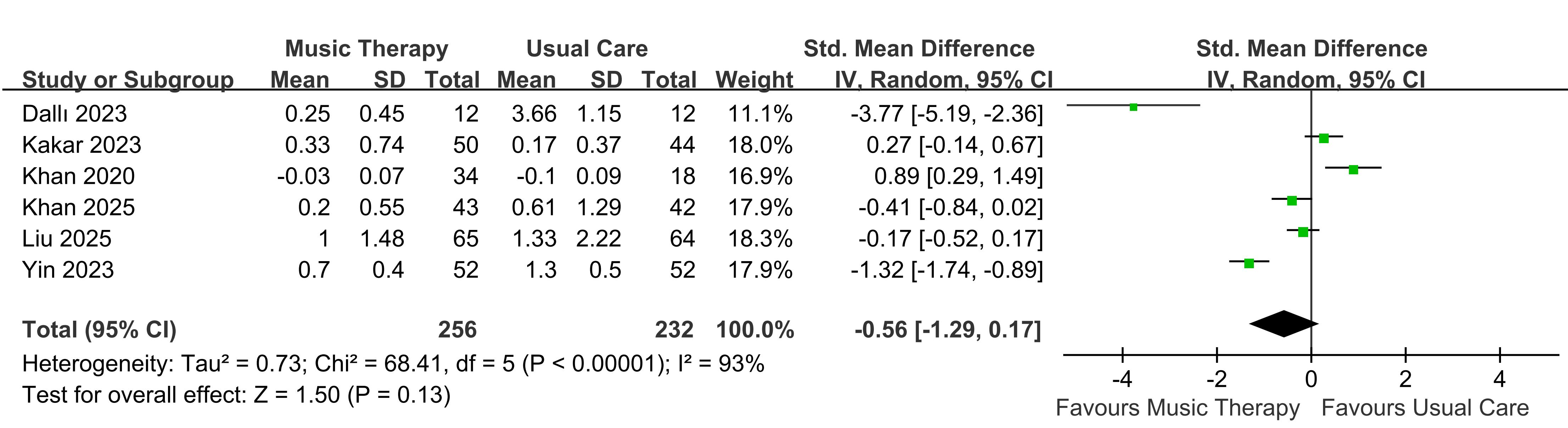


Supplementary Figure S19. Funnel plot for CPOT (pain scores).


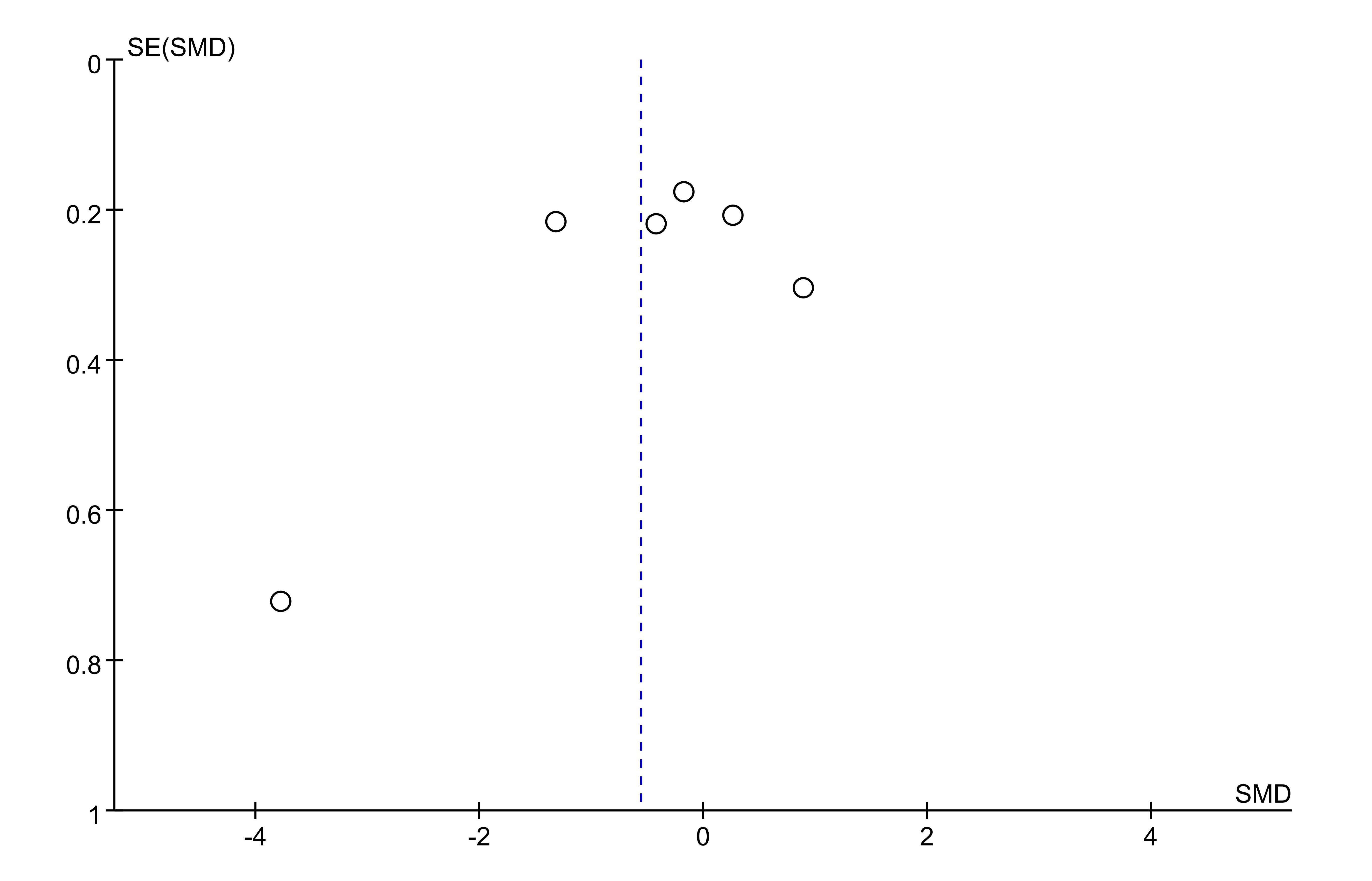

Supplement: Supplementary file 4 [file Supplementary_file_4.docx]
